# Design and validation of a pericentromeric BAC clone set aimed at improving diagnosis and phenotype prediction of supernumerary marker chromosomes

**DOI:** 10.1186/1755-8166-6-45

**Published:** 2013-10-30

**Authors:** Chiara Castronovo, Emanuele Valtorta, Milena Crippa, Sara Tedoldi, Lorenza Romitti, Maria Cristina Amione, Silvana Guerneri, Daniela Rusconi, Lucia Ballarati, Donatella Milani, Enrico Grosso, Pietro Cavalli, Daniela Giardino, Maria Teresa Bonati, Lidia Larizza, Palma Finelli

**Affiliations:** 1 Laboratorio di Citogenetica Medica e Genetica Molecolare, IRCCS Istituto Auxologico Italiano, via Ariosto 13, 20145, Milano, Italy; 2 Servizio di Genetica, A.O. Istituti Ospitalieri, viale Concordia 1, 26100, Cremona, Italy; 3 S.C. Anatomia, Istologia Patologica e Citogenetica, Ospedale Niguarda Ca’ Granda, piazza dell’Ospedale Maggiore 3, 20162, Milano, Italy; 4 S.C.D.U. Genetica Medica, A.O.S. Giovanni Battista, corso Bramante 88, 10126, Torino, Italy; 5 Laboratorio di Genetica Medica, Fondazione IRCCS Ca’ Granda Ospedale Maggiore Policlinico, via Francesco Sforza 28, 20122, Milano, Italy; 6 Clinica Pediatrica 1, Dipartmento di Patofisiologia e Trapianti, Università degli Studi di Milano, Fondazione IRCCS Ca’ Granda Ospedale Maggiore Policlinico, via Francesco Sforza 28, 20122, Milano, Italy; 7 Ambulatorio di Genetica Medica, Ospedale San Luca, IRCCS Istituto Auxologico Italiano, piazzale Brescia 20, 20149, Milano, Italy; 8 Genetica Medica, Dipartimento di Scienze della Salute, Università degli Studi di Milano, via Antonio di Rudinì 8, 20142, Milano, Italy; 9 Dipartmento di Biotecnologie Mediche e Medicina Traslazionale, Università degli Studi di Milano, via Viotti 3/5, 20133, Milano, Italy

**Keywords:** Small supernumerary marker chromosomes, Pericentromeric clone set, Heterochromatin/euchromatin boundary, FISH analysis, Array CGH analysis, Phenotype prediction

## Abstract

**Background:**

Small supernumerary marker chromosomes (sSMCs) are additional, structurally abnormal chromosomes, generally smaller than chromosome 20 of the same metaphase spread. Due to their small size, they are difficult to characterize by conventional cytogenetics alone. In regard to their clinical effects, sSMCs are a heterogeneous group: in particular, sSMCs containing pericentromeric euchromatin are likely to be associated with abnormal outcomes, although exceptions have been reported. To improve characterization of the genetic content of sSMCs, several approaches might be applied based on different molecular and molecular-cytogenetic assays, e.g., fluorescent *in situ* hybridization (FISH), array-based comparative genomic hybridization (array CGH), and multiplex ligation-dependent probe amplification (MLPA).

To provide a complementary tool for the characterization of sSMCs, we constructed and validated a new, FISH-based, pericentromeric Bacterial Artificial Chromosome (BAC) clone set that with a high resolution spans the most proximal euchromatic sequences of all human chromosome arms, excluding the acrocentric short arms.

**Results:**

By FISH analysis, we assayed 561 pericentromeric BAC probes and excluded 75 that showed a wrong chromosomal localization. The remaining 486 probes were used to establish 43 BAC-based pericentromeric panels. Each panel consists of a core, which with a high resolution covers the most proximal euchromatic ~0.7 Mb (on average) of each chromosome arm and generally bridges the heterochromatin/euchromatin junction, as well as clones located proximally and distally to the core. The pericentromeric clone set was subsequently validated by the characterization of 19 sSMCs. Using the core probes, we could rapidly distinguish between heterochromatic (1/19) and euchromatic (11/19) sSMCs, and estimate the euchromatic DNA content, which ranged from approximately 0.13 to more than 10 Mb. The characterization was not completed for seven sSMCs due to a lack of information about the covered region in the reference sequence (1/19) or sample insufficiency (6/19).

**Conclusions:**

Our results demonstrate that this pericentromeric clone set is useful as an alternative tool for sSMC characterization, primarily in cases of very small SMCs that contain either heterochromatin exclusively or a tiny amount of euchromatic sequence, and also in cases of low-level or cryptic mosaicism. The resulting data will foster knowledge of human proximal euchromatic regions involved in chromosomal imbalances, thereby improving genotype–phenotype correlations.

## Background

Small supernumerary marker chromosomes (sSMCs) are additional centric chromosomal segments that are difficult to characterize by conventional cytogenetics alone due to their small size [1]. Excluding the sSMCs associated with five well-known syndromes (Pallister-Killian, isodicentric chromosome 15q [2], isochromosome 18p, Cat Eye [i(22p ~ q)], and derivative chromosome 22 [der(22)t(11;22)] syndromes [1]), the overall risk for a pathological phenotype in prenatal *de novo* cases is 26–30% [3].

The phenotypic expression of sSMCs ranges from asymptomatic to symptomatic, and depends on several factors including chromosomal origin, satellite vs. non-satellite inclusion, euchromatic/heterochromatic content, uniparental disomy (UPD) of the chromosomes homologous to the sSMC, and mosaicism [3]. Furthermore, the presence of centromere-proximal euchromatin on an sSMC correlates with abnormal phenotypes, although several exceptions have been described [4]. Since the optimal strategies for genetic counseling and clinical management depend on the characteristics of sSMCs, it is vitally important to precisely characterize sSMCs in order to obtain additional information regarding their phenotypic effects. To this end, several fluorescent *in situ* hybridization (FISH)-based techniques have been developed over the years [5] for determining the origin of sSMCs and allowing breakpoint characterization, at least in cases of larger euchromatic SMCs. These methods include multicolor FISH (M-FISH) [6], spectral karyotyping (SKY) [7], centromere- and subcentromere-specific M-FISH (cenM-FISH and subcenM-FISH) [3,8,9], multicolor banding [10], and microdissection followed by reverse FISH [11,12]. More recently, a pericentric-ladder-FISH (PCL-FISH) probe set has been developed based on 174 locus-specific BAC probes, and this probe set has been used in dual-color/multicolor–FISH approaches. This tool is specific for the pericentromeric regions and, therefore, enables sSMC breakpoint characterization with a resolution between 1 and ~10 Mb [13].

Furthermore, array-based comparative genomic hybridization (array CGH) analysis has been extensively used in sSMC characterization. This method allows, in a single experiment, determination of the marker chromosomal origin, definition of the size of aberrations (including euchromatic regions), and identification of complex rearrangements or multiple markers in single individuals [14-20]. However, array CGH may fail to identify the origins of very small SMCs in up to 50% of cases because its pericentromeric coverage is limited to the presence of segmental duplications, and it may also be unable to detect low-level and cryptic mosaicism [13,19-21]. Consequently, it is necessary to complement array CGH using FISH approaches [13,22]. In addition, to allow rapid discrimination between sSMCs that are positive or negative for unique sequences, an alternative approach using multiplex ligation-dependent probe amplification (MLPA) analysis has recently been developed for use in the context of prenatal diagnosis [23].

In this study, we report the design and validation of a new pericentromeric Bacterial Artificial Chromosome (BAC) clone set that covers the most proximal euchromatic sequences of all human chromosome arms, as well as the heterochromatin/euchromatin junctions, excluding the short arms of acrocentric chromosomes. This set was designed to improve molecular characterization of sSMCs by FISH analysis, a molecular-cytogenetic technique that, in contrast to array CGH, is available in most cytogenetic laboratories. This new complementary tool will be especially useful in cases of low-level mosaicism and/or very small marker chromosomes, which are likely to consist entirely of heterochromatin or contain only a tiny amount of euchromatic sequence, as demonstrated by some reported sSMC cases.

## Results

### Setting up 43 pericentromeric BAC probe panels

Using FISH analysis, we assayed 561 pericentromeric BAC probes, of which 323 (57.6%) were specific, 163 (29.0%) exhibited multiple cross-hybridizations, and 75 (13.4%) were excluded because of a wrong chromosomal localization. We then established 43 pericentromeric panels (excluding the acrocentric p arms), each of which consists of a high resolution core panel, which bridges the heterochromatin/euchromatin junction and usually comprises 3–7 contiguous or very close clones, as well as clones located proximally and distally to the core (Additional file 1: Table S1). The core panels were completed for 35 chromosome arms (2p, 2q, 3p, 3q, 4p, 4q, 5p, 5q, 6p, 6q, 7p, 7q, 8p, 8q, 9q, 10p, 11q, 12p, 12q, 13q, 14q, 15q, 16p, 16q, 17p, 17q, 18p, 18q, 19p, 19q, 20p, 22q, Xp, Xq, and Yq); they span, generally in a continuum, the proximal ~0.73 Mb (on average) of each chromosome arm (Table 1). A core panel for chromosome Yp was not established because of the lack of a physical map in the reference sequence, whereas the core panels of seven other chromosome arms were incomplete due to either the lack of a heterochromatin/euchromatin junction in the reference sequence (1p, 10q, 11p, 20q, and 21q), or the presence of pericentromeric paralogous segmental duplications (1q and 9p) that prevented us from obtaining unique mapping information (Table 1 and Additional file 1: Table S1).

**Table 1:** Pericentromeric euchromatic coverage of the high-resolution BAC-based core panels

**Chromosome arm**	**Heterochromatic region starting position (Mb)**^***a***^	**Heterochromatic region ending position (Mb)**^***a***^	**Proximal euchromatic BAC clone belonging to the core panel**	**Distance between heterochromatic region and proximal euchromatic core BAC clone (Mb** ***)***^***b***^	**Distal euchromatic BAC clone belonging to the core panel**	**Distance between proximal and distal euchromatic core BAC clones (Mb** ***)***^***c***^
1p^*d*^	121.5		RP11-803J8 at 1p11.2	0.015	CTD-3138A9 at 1p12-p11.2	0.98
1q^*d*^		142.6	RP11-15M9 at 1q21.1	0.76	CTD-2326L14 at 1q21.1	1.46
2p	90.5		CTD-2269O20 at 2p11.2-p11.1	0	RP11-1023H22 at 2p11.2	0.61
2q		96.8	RP11-139J5 at 2q11.1-q11.2	0	RP11-245P4 at 2q11.2	0.96
3p	87.9		RP11-424C9 at 3p11.2-p11.1	0	RP11-598A10 at 3p12.1-p11.2	0.71
3q		93.9	RP11-259L20 at 3q11.1-q11.2	0	RP11-625F19 at 3q11.2	0.87
4p	48.2		RP11-260K18 at 4p12-p11	0	CTD-2057N12 at 4p12	0.88
4q		52.7	RP11-98B6 at 4q11-q12	0	RP11-600M5 at 4q12	0.76
5p	46.1		RP11-10F16 at 5p12-p11	0	RP11-134N5 at 5p12	0.91
5q		50.7	RP11-463E10 at 5q11.1-q11.2	0	CTD-3113M11 at 5q11.2	0.33
6p	58.7		RP11-136G2 at 6p11.2-p11.1	0	RP11-799H20 at 6p11.2	1.02
6q		63.3	RP11-448N11 at 6q11.1-q11.2	0	RP11-78B14 at 6q12	0.30
7p	58.0		CTD-2593N8 at 7p11.2-p11.1	0	RP11-114G11 at 7p11.2	0.66
7q		61.7	CTD-2245O1 at 7q11.1-q11.21	0	RP11-45N18 at 7q11.21	0.84
8p	43.1		RP11-73M19 at 8p11.21-p11.1	0	RP11-577C12 at 8p11.21	0.79
8q		48.1	CTD-2563N10 at 8q11.1-q11.21	0	RP11-367A12 at 8q11.21	0.56
9p^*d*^	47.3		RP11-606B18 at 9p11.2	0.37	RP11-361G22 at 9p11.2	0.64
9q		65.9	CTD-2508M5 at 9q12-q13	0	CTD-2050L17 at 9q13	0.49
10p	38.0		CTD-3195G22 at 10p11.21-p11.1	0	RP11-739D18 at 10p11.21	0.58
10q^*d*^		42.3	RP11-80L2 at 10q11.21	0.08	RP11-351D16 at 10q11.21	1.32
11p^*d*^	51.6		RP11-100E23 at 11p11.12	0.06	RP11-318O24 at 11p11.12	1.00
11q		55.7	CTD-3202L3 at 11q11-q12.1	0	RP11-720L5 at 11q12.1	0.85
12p	33.3		RP11-460N10 at 12p11.21-p11.1	0	RP11-8P13 at 12p11.21	0.48
12q		38.2	RP11-496H24 at 12q11-q12	0	RP11-715M8 at 12q12	0.68
13q		19.5	RP11-294G16 at 13q11-q12.11	0	RP11-71I1 at 13q12.11	0.32
14q		19.1	RP11-639F13 at 14q11.1-q11.2	0	RP11-77E23 at 14q11.2	0.90
15q		20.7	RP11-108C1 at 15q11.1-q11.2	0	RP11-666L22 at 15q11.2	0.34
16p	34.6		RP11-488I20 at 16p11.2-p11.2	0	RP11-1088B6 at 16p11.2	0.32
16q		47.0	RP11-627O2 at 16q11.2-q12.1	0	RP11-671L23 at 16q12.1	0.36
17p	22.2		RP11-718K3 at 17p11.2-p11.1	0	RP11-937K3 at 17p11.2	0.51
17q		25.8	RP11-1049N9 at 17 q11.1-q11.2	0	RP11-59G20 at 17q 11.2	0.52
18p	15.4		RP11-1133K23 at 18p11.21-p11.1	0	RP11-1025M21 at 18p11.21	0.52
18q		19.0	RP11-746M23 at 18q11.1-q11.2	0	RP11-60G3 at 18q11.2	0.66
19p	24.4		RP11-350E11 at 19p12-p11	0	RP11-642I13 at 19p12	0.86
19q		28.6	CTD-2045N7 at 19q11-q12	0	RP11-347I6 at 19q12	0.95
20p	25.6		RP11-161K13 at 20p11.21-p11.1	0	RP11-156D15 at 20p11.21	0.49
20q^*d*^		29.4	CTD-2311M18 at 20q11.21	0.02	RP11-1147I19 at 20q11.21	1.17
21q^*d*^		14.3	RP11-203F20 at 21q11.2	0.13	RP11-1025M7 at 21q11.2	0.93
22q		17.9	RP11-958H20 at 22q11.1-q11.21	0	RP11-81B3 at 22q11.21	0.71
Xp	58.1		CTD-2225J11 at Xp11.21-p11.1	0	RP11-936C8 at Xp11.21	0.96
Xq		63.0	RP11-943J20 at Xq11.1-q11.2	0	RP11-284B18 at Xq11.2	0.54
Yp^*e*^	11.6		RP11-108I14 at Yp11.2	1.50		
Yq		13.4	RP11-1100G7 at Yq11.1-q11.21	0	RP11-91N9 at Yq11.21	1.1

^*a*^The location of the heterochromatic region is an approximation taken from the February 2009 release of the UCSC Human Genome Browser Database, which indicates the distance from the most distal available p-arm sequence to the centromere start and end for that chromosome. For chromosomes 1q, 9q, and 16q, the heterochromatic region ending position indicates the telomeric border of the heterochromatic bands 1q12, 9q12, and 16q11.2, respectively.

^*b*^The distance between the heterochromatic region and the most proximal available euchromatic BAC clone indicates the starting position of the pericentromeric coverage of the BAC core panel for each chromosome arm.

^*c*^The distance between the proximal and distal euchromatic core BAC clones indicates the pericentromeric euchromatic coverage of the core panel for each chromosome arm; if the proximal BAC clone overlaps with heterochromatin/euchromatin bridge, the distance refers only to the euchromatic sequence.

^*d*^The core panels of chromosomes 1p, 10q, 11p, 20q, and 21q were not completed because of the lack of heterochromatin/euchromatin-bridging physical maps in the reference sequence. The core panels of chromosomes 1q and 9p are incomplete due to the presence of pericentromeric paralogous segmental duplications; therefore, the corresponding core panel probes were excluded from the clone set because their hybridization signals do not give unique mapping information. The partial core panel of chromosome 10q was established using non-contiguous clones.

^*e*^The core panel of chromosomes Yp was not established because of the lack of a pericentromeric physical map in the reference sequence.

### Molecular-cytogenetic characterization of 19 sSMCs

The utility of the clone set was then validated by the molecular characterization of 19 sSMCs, six of which were ascertained during routine prenatal testing (Additional file 2: Table S2). Nine sSMCs (~47%) were derived from acrocentric chromosomes, six (~32%) were inherited (patients 6, 8, 10–13), of which two were detected in two siblings (patients 10 and 11) and are complex marker chromosomes originating from a maternal balanced rearrangement. Multiple markers occurred in a single adult patient (5%) (patient 15). Mosaicism was detected in seven patients (~37%) (patients 1–5, 15, and 18), in most cases involving non-acrocentric chromosomes. Uniparental disomy (UPD) analysis of sSMC sister chromosomes was not performed. Details of sSMC characterization are provided in Additional file 2: Table S2.

**Figure 1:**
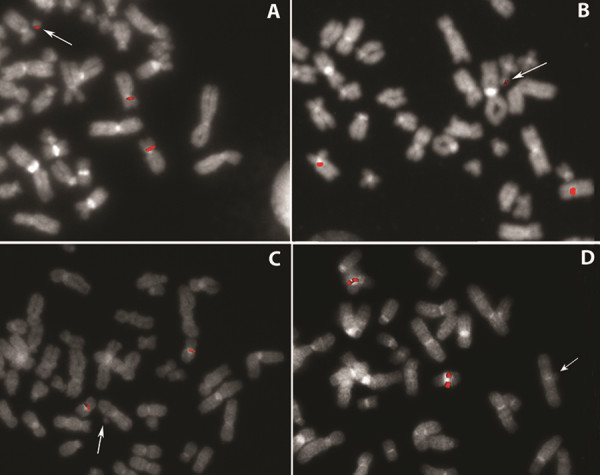
**FISH characterization of sSRC(10) of patient 4 (A, B) and sSRC(16) of patient 14 (C, D), including breakpoint mapping and sizing of the euchromatic region. (A)** Partial metaphase hybridized with the 10p11.23 BAC probe RP11-39E10, showing signals on both chromosome 10 homologs and the der(10) marker chromosome (arrow). **(B)** Partial metaphase hybridized with the BAC probe RP11-178A10 at 10q11.21, showing a diminished hybridization signal on the der(10) marker chromosome (arrow) relative to the chromosome 10 homologs. **(C and D)** Partial metaphases hybridized with the BAC probes RP11-488I20 at 16p **(C)** and CTD-2382P11 at 16q **(D)**, showing signals on the chromosome 16 homologs but not sSRC(16) (arrows).

**Figure 2:**
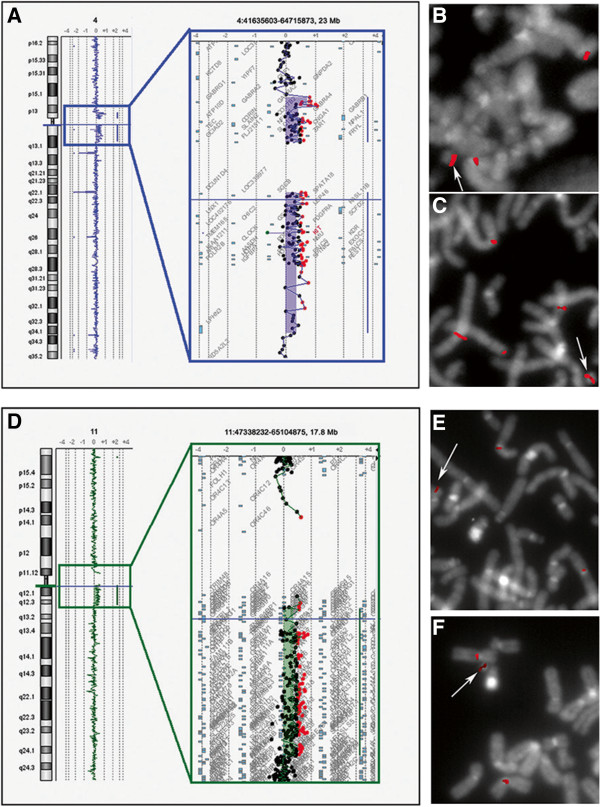
**Combined array-CGH and FISH characterization of SRC(4) of patient 3 (A-C) and SRC(11) of patient 5 (D-F).** Upper row: **(A)** Identification of a heterozygous duplication of 15.4 Mb (chr4:45,754,992–61,126,608, hg19) using array CGH (Agilent 4 × 44K) in patient 3. The BAC probes RP11-178N2 at 4p12 **(B)** and RP11-91C3 at 4q13.1 **(C)** produce hybridization signals on both chromosomes 4 and the SRC(4). Bottom row: **(D)** Identification of a heterozygous duplication of 13.4 Mb (chr11:50,378,743–63,743,029, hg19) using array CGH (Agilent 4 × 44K) in patient 5. The BAC probes RP11-746P9 at 11p11.12 **(E)** and RP11-872D17 at 11q12 **(F)** produce hybridization signals on both chromosomes 11 and the SRC(11).

**Figure 3:**
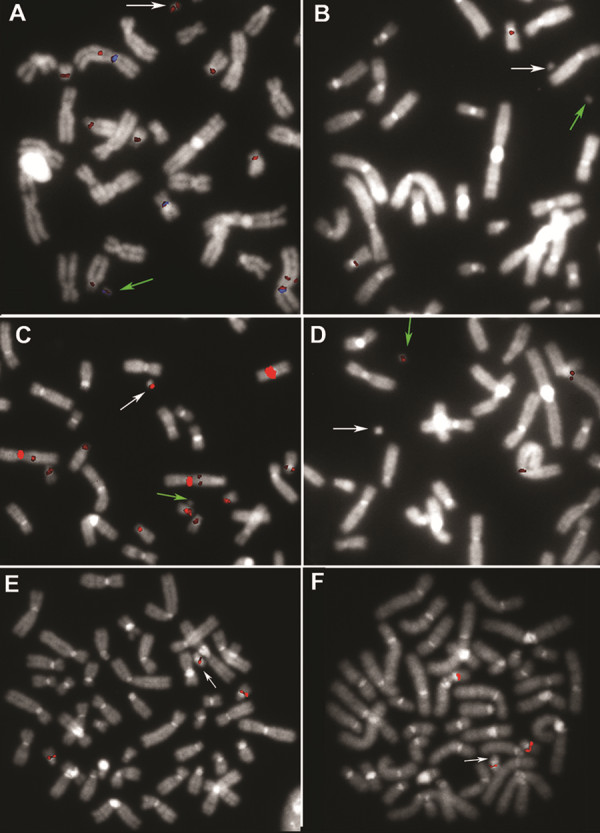
**FISH characterization of sSMC(2) and sSMC(18) of patient 15 (A-D), and sSMC(22) of patient 18 (E, F). (A)** Partial metaphase hybridized with the BAC probe RP11-749K13 at 18p11.21 (red), showing a hybridization signal on idic(18;18) marker chromosomes (white arrow) that is enlarged relative to those on chromosome 18 homologs, as well as cross-hybridization signals on the der(2) marker chromosome (green arrow) and chromosome 2 homologs. The BAC probe RP11-1069D4 at 2p11.1 (blue) produces hybridization signals on der(2) marker chromosome (green arrow) and chromosome 2 homologs. **(B)** Partial metaphase hybridized with the BAC probe RP11-1035O2 at 18q11.1 does not show a hybridization signal on idic(18;18) (white arrow). **(C)** Partial metaphase hybridized with the BAC probe RP11-134N21 at 2q11.1, which is deleted on der(2) (green arrow), showing cross-hybridization signals on both idic(18;18) (white arrow) and chromosome 18 homologs. **(D)** Partial metaphase hybridized with the BAC probe RP11-71B7 at 2q11.1, showing a hybridization signal on der(2) (green arrow) and on chromosome 2 homologs. **(E)** Metaphase hybridized with the BAC probes RP11-1053O2 at 22q11.21 shows an equal signal on the idic(22;22) marker chromosome (arrow) relative to the signals on the chromosome 22 homologs. **(F)** Metaphase hybridized with the BAC probe RP11-690P21 at 22q11.21 shows a diminished signal on the idic(22;22) marker chromosome (arrow) relative to the signals on the chromosome 22 homologs.

Using the core panel probes, we were able to rapidly distinguish between heterochromatic (1/19) and pericentromeric euchromatic marker chromosomes (11/19), even in cases of low-level mosaicism. In addition, we either precisely established or estimated the size of the euchromatic content in 17 out of 19 sSMCs (Additional file 2: Table S2, Figures 1, 2 and 3). The euchromatic DNA present on the sSMCs ranged from ~0.13 Mb to more than 10 Mb (Additional file 2: Table S2). However, sSMC characterization was not completed in patient 6, due to the incompleteness of the chromosome 21q core panel physical map in the reference sequence, and in patients 7–11 and 15, due to sample insufficiency (Additional file 2: Table S2).

In two patients, we detected large marker chromosomes in high-level mosaicism, as follows: a ring chromosome 4 [r(4)] in patient 3, and an r(11) in patient 5 (Additional file 2: Table S2, Figure 2). The molecular characterization of these SMCs was performed using the panel probes and subsequently refined by array CGH analysis, which allowed a more precise definition of the breakpoints. In particular, the chromosome 4p breakpoint was mapped between BAC probes RP11-89F4 and RP11-178N2 (chr4:44,621,297 − 45,736,237, hg19) (Figure 2B), and then narrowed down by array CGH to a 236 kb region between oligonucleotide probes A_14_P127561 and A_14_P128650 (chr4:45,518,972 − 45,754,992, hg19); thus, the breakpoint was mapped at ~45.5–45.7 Mb from the 4p telomere (chr4:45,518,972–45,736,237, hg19) (Additional file 2: Table S2, Figure 2A). Similarly, the 4q breakpoint was mapped between BACs RP11-91C3 (Figure 2C) and RP11-63E13 (chr4:61,086,318–66,600,553, hg19), and the position was then refined to a location between oligonucleotides A_14_P137150 and A_14_P133582 (chr4:61,126,608–61,343,360, hg19) (Additional file 2: Table S2, Figure 2A). For sSRC(11), the 11p breakpoint was mapped between BACs RP11-1062A8 and RP11-746P9 (chr11:49,259,387–50,111,641, hg19) (Figure 2E), and then refined between oligonucleotides A_14_P126799 and A_14_P134986 (chr11:49,919,441–50,378,743, hg19); thus, this breakpoint mapped precisely at 49.9 − 50.1 Mb from the 11p telomere (chr11:49,919,441–50,111,641, hg19) (Additional file 2: Table S2, Figure 2D). The 11q breakpoint was characterized only by array CGH because the 11q panel probes were not informative due to their proximal position (Additional file 2: Table S2, Figure 2D,F). No further cryptic genomic imbalances were detected in either patient.

## Discussion

Pericentromeric regions of human chromosomes are transitional territories between centromeric heterochromatin and euchromatic regions. They represent complex mosaic structures, including coding sequences interspersed with non-coding sequences [24]. Therefore, sequencing of these regions is technically difficult, and a complementary approach is necessary to clarify their role in human disease.

sSMCs generally contain a centromeric/pericentromeric region, and their precise characterization is a powerful tool for identifying which genomic regions lead to abnormalities when they are affected by dosage imbalances. Over the years, numerous FISH-based approaches have been developed, and these methods have contributed to improvements in sSMC characterization. However, assays such as M-FISH/SKY [6,7], cenM-FISH [8] and subcenM-FISH [3,9] are limited to the identification of the sSMC chromosomal origin or the characterization of larger euchromatic SMCs, and only the use of FISH-banding or locus-specific probes [13,25,26] can improve breakpoint characterization [13,27,28]. In particular, the PCL-FISH probe set, recently developed by Hamid et al., (2012) [13], is a bar-code FISH assay that constitutes a 10 Mb raster along pericentromeric chromosomal regions, allowing the determination of mosaic and non-mosaic sSMC breakpoints within genomic regions of 1–10 Mb in size. In addition, this approach has been particularly useful in characterizing cryptic mosaic sSMCs [29], and for easily defining all involved breakpoints [13]. However, because it covers the most proximal 1 Mb of euchromatic sequences of each chromosome arm with a very low resolution, PCL-FISH is not useful for the characterization of very small SMCs that contain only tiny amounts of euchromatic sequence [13]. Likewise, a more sensitive technique such as array CGH, which can significantly narrow down sSMC breakpoints [14-20,30], can still yield incomplete pericentromeric coverage due to the presence of large duplicated sequences. Moreover, array CGH cannot detect low-level and/or cryptic mosaic sSMCs.

Therefore, in order to provide a complementary tool for sSMC characterization, we established a FISH-based pericentromeric BAC clone set, including probes that cover the most proximal euchromatic ~0.7 Mb (on average) of each chromosome arm at a high resolution. The pericentromeric probe set also includes probes that bridge the heterochromatin/euchromatin junctions (Table 1 and Additional file 1: Table S1), enabling rapid discrimination between euchromatic (11/19 in the present series) and heterochromatic (1/19) sSMCs, irrespective of marker chromosome origin (Additional file 2: Table S2). The most proximal probes were chosen independently of the presence of segmental duplications, with the exceptions of chromosomes 1q and 9p, where pericentromeric paralogous segmental duplications have been detected. As expected, a significant percentage of the assayed probes (~13%) were mislocalized, supporting the need for the large screening effort we performed to verify the predicted physical position of each clone. As previously reported for PCL-FISH [13], we confirmed the utility of our new BAC-probe set in characterizing low-level mosaic sSMCs (Additional file 2: Table S2), suggesting that this approach could be applied to breakpoint identification in cases of cryptic mosaic sSMCs; however, no pertinent cases are present in the series reported here.

In terms of genotype–phenotype correlation, the data we collected regarding sSMC characterization allowed us to confirm the existence of pericentromeric euchromatic critical and noncritical regions surrounding the centromeres of all chromosome arms, i.e., regions in which trisomy or tetrasomy either does or does not correlate with pathological phenotypes [4]. Accordingly, we declare no clinical signs reported in association with acrocentric sSMCs that have breakpoints localized in predicted pericentromeric noncritical regions [4,14,16,22,31] (patients 6–9, 12, and 13), with the exception of patient 7, who exhibited a growth delay likely not associated with the sSMC (Additional file 2: Table S2). Furthermore, in siblings 10 and 11, the 14q breakpoint characterization was useful in linking the reported clinical findings specifically to trisomy of 6pter-p25 (Additional file 2: Table S2). By contrast, we classified the larger idic(22;22) marker chromosomes (patients 16–18) as type I Cat Eye Syndrome (CES) chromosomes, which results in the CES phenotype [32], as confirmed in patient 17 (Additional file 2: Table S2). In addition, in patient 18, the molecular characterization of idic(22;22) revealed asymmetrical breakpoints that resulted in both a tetrasomy of ~750 kb, which featured the gap reported between the end of the noncritical region and the start of the critical region [4], and a trisomy of ~150–400 kb of euchromatic sequences that are included within the 22q predicted critical region [4] (Additional file 2: Table S2, Figure 3E,F). These observations suggest that the mild phenotype of patient 18, relative to the classical CES clinical presentation, can be at least partially attributed to trisomy rather than tetrasomy of the same euchromatic region, combined with low-level mosaicism of sSMC(22) (Additional file 2: Table S2).

Among the non-acrocentric marker chromosomes collected in our series, the SMC characterization revealed breakpoints within pericentromeric noncritical regions in two cases [4,14-16,20,22,33,34], consistent with the observation that the corresponding patients (3 and 15) had normal phenotypes (Figures 2A–C and 3A–D, Additional file 2: Table S2). Notably, the association of sSMC(2) and sSMC(18) observed in patient 15 has not been previously reported. By performing FISH analysis, we identified pericentromeric segmental duplications shared by chromosomes 2 and 18 (Figure 3A–D), and hypothesized that a complex genomic rearrangement had occurred between those two chromosomes, resulting in sSMC formation and in the simultaneous deletion of the sequence covered by probe RP11-134N21 from sSMC(2) (Figure 3D, Additional file 2: Table S2).

In regard to pathological non-acrocentric sSMCs, our molecular characterization helped us to either refine or confirm the boundaries of the predicted critical regions and to improve genotype–phenotype correlations. For example, characterization of patient 2’s mosaic sSRC(1) indicated possible trisomy of 1p12–q21.1 chromosomal region, involving at most ~1.91 Mb of euchromatic sequences at 1q21.1 (Additional file 2: Table S2). Although the boundaries between the critical and noncritical regions on 1q are not yet available [4], we propose that trisomy of 1q21.1 might be responsible for patient 2’s phenotype (Additional file 2: Table S2), as suggested by previous data [3,9,35-38]. However, neurological abnormalities like those exhibited by our patient have been previously reported in patients carrying mosaic sSMC(1), resulting in trisomy of the 1p12–q12 region. In those cases, the 1q breakpoints were mapped within the 1q12 heterochromatic region, suggesting that the 1p trisomy was responsible for the pathological phenotype [3,8,9,39].

Both breakpoints of patient 5’s SRC(11) were inferred to have occurred in the pericentromeric critical regions, leading to mosaic trisomy of the 11p11.12–q13.1 region (Additional file 2: Table S2, Figure 2E,F). To date, only one patient (11-W-p11.12/3-1 in the sSMC database [4]) has been reported to have a mosaic sSRC(11) characterized by breakpoints mapped within the same chromosomal bands found in our patient [4]. The reported pathological clinical presentation, characterized by dysmorphism and severe developmental delay, resembles that observed in patient 5 (Additional file 2: Table S2), confirming the pathogenetic role of mosaic trisomy of 11p11.12–q13.1.

Finally, sSMC(10) of patient 4 and sSRC(16) of patient 14 were both ascertained during prenatal testing. The sSRC(16) exclusively involved centromeric heterochromatin (Additional file 2: Table S2, Figure 1C, D), leading the parents to continue the pregnancy, which ended with the birth of a normal baby. By contrast, the sSMC(10) resulted in mosaic trisomy of the 10p11.23–q11.21 region (Additional file 2: Table S2, Figure 1A,B). We considered that a pathological phenotype might arise due to trisomy of at least 3.6 Mb of 10p proximal euchromatic sequences because the 10q breakpoint mapped in the predicted noncritical region [4,17,40]. This hypothesis was subsequently confirmed by the patient’s neonatal phenotype (Additional file 2: Table S2).

## Conclusions

To summarize, our data demonstrate the potential value of our pericentromeric clone set for characterization of sSMCs in both prenatal and postnatal diagnostics. Due to the fact that the established resource covers all available pericentromeric regions, it may be particularly useful in cases of very small marker chromosomes, allowing rapid discrimination between heterochromatic and euchromatic sSMCs, as well as precise sizing of imbalances. We also demonstrated the complementarity of FISH analysis using the pericentromeric clone set with array CGH analysis in the characterization of large marker chromosomes.

Apart from ad hoc combinations of different methods, performed when requested, FISH analysis is currently the only available technique for analyzing sSMCs in low or cryptic mosaicism. However, to use this probe set in prenatal cases, cytogenetic labs need to have sufficient resources to store the BAC clones and prepare the probes within a short time. Therefore, although application of this tool in prenatal diagnosis would be beneficial, we strongly recommend that it is used in research aimed at increasing our knowledge of the imbalances of human proximal euchromatic regions, thereby improving genotype–phenotype correlations and the assessment of the genetic risks of supernumerary marker chromosomes.

## Methods

### sSMC samples

Chromosomal samples from peripheral blood lymphocytes and amniotic fluid were collected from five cytogenetic labs. All samples were previously karyotyped by conventional cytogenetics (Q- or G-banding), and the sSMC origin was previously identified by FISH analysis using commercial centromere-specific probes (Vysis, Maidenhead, UK), following the manufacturer’s instructions. In both cases, at least 16 metaphases were analyzed.

### FISH analysis

RPCI-11 and CTD BAC clones covering the pericentromeric genomic regions of human chromosome arms were selected by consulting the UCSC Genome Browser Database (http://genome.cse.ucsc.edu, hg19, February 2009). Clones were provided by Prof. Mariano Rocchi, University of Bari (IT) and Invitrogen (Carlsbad, CA, USA). The probes’ physical positions were verified on a few control metaphases derived from peripheral blood lymphocytes. All probes were labeled by nick-translation with biotin (Hoffman-La Roche, Basel, Switzerland), digoxigenin (Hoffman-La Roche), or Cy3 (Amersham Biosciences, Chalfont St. Giles, UK), and then visualized using FITC–anti-digoxigenin antibodies (Hoffman-La Roche) or streptavidin-DEAC (Sigma-Aldrich, St. Louis, MO, USA). Image acquisition was performed on a Leica DMRA2 fluorescence microscope (Leica, Wetzlar, Germany) equipped with Leica filters specific for DAPI, FITC, Cy3, and DEAC. Images were acquired using a charge-coupled device (CCD) camera (Leica) with a magnification factor of 100×. Image analysis was performed using the Leica CW4000-FISH software (version Y1.3.1). In the initial step of sSMC characterization, the most proximal available core probe(s) for the involved chromosome arm(s) was used to discriminate between euchromatic and heterochromatic sSMCs. Next, fine breakpoint mapping was performed using clones within and/or distal to the core panels, depending on sSMC size. A single-color, dual-color, or three-color hybridization approach was chosen depending on the available amount of sSMC sample and the time available to complete the analysis. In case of non-mosaic sSMCs, at least 16 metaphases were analyzed, whereas in cases of mosaic sSMCs, the number of analyzed metaphases decreased proportionally with the level of mosaicism. The FISH protocols of Lichter et al. [41] and Lichter and Cremer [42] were followed, with minor modifications.

### Array CGH analysis

Array CGH analysis was performed using the Human Genome CGH Microarray Kit 4 × 44K (Agilent Technologies, Palo Alto, CA), which consists of 42,494 60-mer oligonucleotide probes covering the entire genome with an average spatial resolution of ~43 kb. From both test and sex-matched reference (Promega, Southampton, UK) samples, 3 μg of genomic DNA, previously extracted from probands’ whole blood using the GenElute^TM^ Blood Genomic DNA kit (Sigma-Aldrich), was processed according to the manufacturer’s protocol. Images were obtained using the Agilent Feature Extraction software (version 9.1), and chromosomal profiles were obtained using the ADM-2 algorithm provided by DNA Analytics software (v4.0) (Agilent Technologies). 

## Consent

Written informed consent to the research investigation, which was approved by the Ethical Clinical Research Committee of Istituto Auxologico Italiano, was obtained from either the adult patients or one of the parents in case of child patients.

## Abbreviations

Array CGH: Array comparative genomic hybridization; CenM-FISH: Centromere-specific M-FISH; FISH: Fluorescence in situ hybridization; M-FISH: Multcolor-FISH; PCL-FISH: Pericentric-ladder-FISH; SKY: Spectral karyotyping; sSMC: Small supernumerary marker chromosomes; SubcenM-FISH: Subcentromere-specific M-FISH; UPD: Uniparental disomy.

## Competing interests

The authors declare that they have no competing interests.

## Authors’ contributions

CC: study design, acquisition, analysis and interpretation of data from FISH and array-CGH analyses; manuscript preparation; VE: acquisition, analysis and interpretation of data from FISH analysis, review and approval of the manuscript final version; CM: acquisition, analysis and interpretation of data from array-CGH, review and approval of the manuscript final version; TS: sample collection, karyotyping and sSMC origin characterization, review and approval of the manuscript final version; RL: sample and clinical data collection, karyotyping and sSMC origin characterization, review and approval of the manuscript final version; AMC: sample collection, karyotyping and sSMC origin characterization, review and approval of the manuscript final version; GS: sample collection, karyotyping and sSMC origin characterization, review and approval of the manuscript final version; RD: acquisition, analysis and interpretation of data from array-CGH; review and approval of the manuscript final version; BL: sample collection, karyotyping and sSMC origin characterization, review and approval of the manuscript final version; MD: clinical data collection, review and approval of the manuscript final version; GE: clinical data collection, review and approval of the manuscript final version; CP, clinical data collection, review and approval of the manuscript final version; GD, sample collection, karyotyping and sSMC origin characterization, review and approval of the manuscript final version; BMT, clinical data collection, review and approval of the manuscript final version; LL: study design, critical revision of the manuscript, approval of the final version; FP: study design, interpretation of experimental data, critical revision of the manuscript, approval of the final version. All authors read and approved the final manuscript.

## Authors’ information

CC: biologist, specialist in Medical Genetics, PhD degree in Experimental Pathology and Neuropathology, contract researcher at the Medical Cytogenetics and Molecular Genetics Lab, Istituto Auxologico Italiano, Milano, Italy;

VE: biologist, specialist in Medical Genetics, currently fixed-term contract at the Cytogenetics Lab, Ospedale Niguarda Ca’ Granda, Milano, Italy;

CM: biotechnologist, student of the School of Specialty in Medical Genetics, contract researcher at the Medical Cytogenetics and Molecular Genetics Lab, Istituto Auxologico Italiano, Milano, Italy;

TS: biologist, specialist in Medical Genetics, permanent position at the Cytogenetics Lab, Istituti Ospitalieri, Cremona, Italy;

RL: biologist, specialist in Medical Genetics, manager at the Pre-and Post-Natal Diagnostic Unit of the Cytogenetics laboratory, Niguarda Ca’ Granda Hospital, Milano, Italy;

AMC: biologist, specialist in Medical Genetics, permanent position at the Cytogenetics Lab, S. Giovanni Battista Hospital, Torino, Italy;

GS: biologist, specialist in Medical Genetics, head of the Cytogenetics Lab, Fondazione IRCCS Ca’ Granda Ospedale Maggiore Policlinico, Milano, Italy;

RD: biotechnologist, specialist in Medical Genetics, currently post-doc fellowship at the Medical Genetics lab, Department of Health Sciences, University of Milan;

BL: biologist, specialist in Medical Genetics, permanent position at the Medical Cytogenetics and Molecular Genetics Lab of Istituto Auxologico Italiano, Milano, Italy;

MD: medical doctor, specialist in Medical Genetics, permanent position at the Paediatric Unit, Fondazione IRCCS Ca’ Granda Ospedale Maggiore Policlinico, Milano, Italy;

GE: medical doctor, specialist in Medical Genetics, permanent position at the Division of Medical Genetics, S. Giovanni Battista Hospital, Torino, Italy;

CP: medical doctor, specialist in Medical Genetics, permanent position at the Division of Medical Genetics, Istituti Ospitalieri, Cremona, Italy;

GD: biologist, specialist in Medical Genetics, head of the Cytogenetics Diagnostic Unit, Medical Cytogenetics and Molecular Genetics Lab, Istituto Auxologico Italiano, Milano, Italy;

BMT: medical doctor, specialist in Medical Genetics, permanent position at the Division of Medical Genetics, San Luca Hospital, Istituto Auxologico Italiano, Milano, Italy;

LL: medical doctor, specialist in Medical Genetics, professor of Medical Genetics, Department of Health Sciences, University of Milano; research director of the Medical Cytogenetics and Molecular Genetics Lab, Istituto Auxologico Italiano, Milano, Italy;

FP: biologist, specialist in Medical Genetics, PhD degree in Genetics and Molecular Evolution, associate professor of Medical Genetics, Department of Medical Biotechnology and Translational Medicine, University of Milano; head of the Research Unit, Medical Cytogenetics and Molecular Genetics Lab, Istituto Auxologico Italiano, Milano, Italy.

## Supplementary Material

Additional file 1: Table S1 Detailed list of the collected 486 pericentromeric BAC probes, including the 214 clones which belong to the high-resolution core panels, according to the UCSC Genome Browser Database assembly hg19. Click here for file

Additional file 2: Table S2 Summary of 19 sSMCs characterized using the pericentromeric BAC clone set, including clinical data of the carrying patients. Click here for file

## References

[B1] Liehr T Claussen U Starke H Small supernumerary marker chromosomes (sSMC) in humans Cytogenet Genome Res 2004 107 55 67 10.1159/000079572 15305057

[B2] Battaglia A The inv dup (15) or idic (15) syndrome (tetrasomy 15q) Orphanet J Rare Dis 2008 3 30 10.1186/1750-1172-3-30 19019226 PMC2613132

[B3] Liehr T Mrasek K Weise A Dufke A Rodríguez L Martínez Guardia N Sanchís A Vermeesch JR Ramel C Polityko A Haas OA Anderson J Claussen U von Eggeling F Starke H Small supernumerary marker chromosomes-progress towards a genotype-phenotype correlation Cytogenet Genome Res 2006 112 23 34 10.1159/000087510 16276087

[B4] Liehr T Small supernumerary marker chromosomes (sSMC) Institute of Human Genetics and Anthropology, University of Jena http://www.med.uni-jena.de/fish/sSMC/00START.htm. Accessed 08/08/2013

[B5] Liehr T Basics and literature on multicolor fluorescence in situ hybridization application http://www.fish.uniklinikum-jena.de/mFISH.html

[B6] Speicher MR Gwyn Ballard S Ward DC Karyotyping human chromosomes by combinatorial multi-fluor FISH Nat Genet 1996 12 368 375 10.1038/ng0496-368 8630489

[B7] Schröck E du Manoir S Veldman T Schoell B Wienberg J Ferguson-Smith MA Ning Y Ledbetter DH Bar-Am I Soenksen D Garini Y Ried T Multicolor spectral karyotyping of human chromosomes Science 1996 273 494 497 10.1126/science.273.5274.494 8662537

[B8] Nietzel A Rocchi M Starke H Heller A Fiedler W Wlodarska I Loncarevic IF Beensen V Claussen U Liehr T A new multicolor-FISH approach for the characterization of marker chromosomes: centromere-specific multicolor-FISH (cenM-FISH) Hum Genet 2001 108 199 204 10.1007/s004390100459 11354630

[B9] Starke H Nietzel A Weise A Heller A Mrasek K Belitz B Kelbova C Volleth M Albrecht B Mitulla B Trappe R Bartels I Adolph S Dufke A Singer S Stumm M Wegner RD Seidel J Schmidt A Kuechler A Schreyer I Claussen U von Eggeling F Liehr T Small supernumerary marker chromosomes (SMCs): genotype-phenotype correlation and classification Hum Genet 2003 114 51 67 10.1007/s00439-003-1016-3 13680362

[B10] Liehr T Starke H Heller A Kosyakova N Mrasek K Gross M Karst C Steinhaeuser U Hunstig F Fickelscher I Kuechler A Trifonov V Romanenko SA Weise A Multicolor fluorescence in situ hybridization (FISH) applied to FISH-banding Cytogenet Genome Res 2006 114 240 244 10.1159/000094207 16954660

[B11] Engelen JJ Albrechts JC Hamers GJ Geraedts JP A simple and efficient method for microdissection and microFISH J Med Genet 1998 35 265 268 10.1136/jmg.35.4.265 9598716 PMC1051270

[B12] Liehr T Heller A Starke H Rubtsov N Trifonov V Mrasek K Weise A Kuechler A Claussen U Microdissection based high resolution multicolour banding for all 24 human chromosomes Int J Mol Med 2002 9 335 339 11891523

[B13] Hamid AB Kreskowski K Weise A Kosayakova N Mrasek K Voigt M Santos Guilherme R Wagner R Hardekopf D Pekova S Karamysheva T Liehr T Klein E How to narrow down chromosomal breakpoints in small and large derivative chromosomes – a new probe set J Appl Genetics 2012 53 259 269 10.1007/s13353-012-0098-9 22544657

[B14] Baldwin EL May LF Justice AN Martin CL Ledbetter DH Mechanisms and consequences of small supernumerary marker chromosomes: from barbara McClintock to modern genetic-counselling issues Am J Hum Genet 2008 82 398 410 10.1016/j.ajhg.2007.10.013 18252220 PMC2427313

[B15] Ballif BC Rorem EA Sundin K Lincicum M Gaskin S Coppinger J Kashork CD Shaffer LG Bejjani BA Detection of low-level mosaicism by array CGH in routine diagnostic specimens Am J Med Genet A 2006 140 2757 2767 17103431 10.1002/ajmg.a.31539

[B16] Ballif BC Hornor SA Sulpizio SG Lloyd RM Minier SL Rorem EA Theisen A Bejjani BA Shaffer LG Development of a high-density pericentromeric region BAC clone set for the detection and characterization of small supernumerary marker chromosomes by array CGH Genet Med 2007 9 150 162 10.1097/GIM.0b013e3180312087 17413419

[B17] Sheth F Andrieux J Ewers E Kosyakova N Weise A Sheth H Romana SP LeLorc’h M Delobel B Theisen O Liehr T Nampoothiri S Sheth J Characterization of sSMC by FISH and molecular techniques Eur J Med Genet 2011 54 247 255 10.1016/j.ejmg.2011.01.011 21316495

[B18] Vetro A Manolakos E Petersen MB Thomaidis L Liehr T Croci G Franchi F Marinelli M Meneghelli E Dal Bello B Cesari S Iasci A Arrigo G Zuffardi O Unexpected results in the constitution of small supernumerary marker chromosomes Eur J Med Genet 2012 55 185 190 10.1016/j.ejmg.2012.01.010 22342433

[B19] Reddy KS Aradhya S Meck J Tiller G Abboy S Bass H A systematic analysis of small supernumerary marker chromosomes using array CGH exposes unexpected complexity Genet Med 2013 15 3 13 10.1038/gim.2012.78 22935720

[B20] Marle N Martinet D Aboura A Joly-Helas G Andrieux J Flori E Puechberty J Vialard F Sanlaville D Fert Ferrer S Bourrouillou G Tabet A Quilichini B Simon-Bouy B Bazin A Becker M Stora H Amblard S Doco-Fenzy M Molina Gomes D Girard-Lemaire F Cordier M Satre V Schneider A Lemeur N Chambon P Jacquemont S Fellmann F Vigouroux-Castera A Molignier R Molecular characterization of 39 *de novo* sSMC: contribution to prognosis and genetic counseling, a prospective study Clin Genet 2013 10.1111/cge.12138. [Epub ahead of print] 10.1111/cge.12138 23489061

[B21] Bui TH Vetro A Zuffardi O Shaffer LG Current controversies in prenatal diagnosis 3: is conventional chromosome analysis necessary in the post-array CGH era? Prenat Diagn 2011 31 235 243 10.1002/pd.2722 21374637

[B22] Manolakos E Kefalas K Neroutsou R Lagou M Kosyakova N Ewers E Ziegler M Weise A Tsoplou P Rapti SM Papoulidis I Anastasakis E Garas A Sotiriou S Eleftheriades M Peitsidis P Malathrakis D Thomaidis L Kitsos G Orru S Liehr T Petersen MB Kitsiou-Tzeli S Characterization of 23 small supernumerary marker chromosomes detected at pre-natal diagnosis: the value of fluorescence in situ hybridization Mol Med Rep 2010 3 1015 1022 21472348 10.3892/mmr.2010.358

[B23] Van Opstal D Boter M Noomen P Srebniak M Hamers G Galjaard RJ Multiplex ligation dependent probe amplification (MLPA) for rapid distinction between unique sequence positive and negative marker chromosomes in prenatal diagnosis Mol Cytogenet 2011 4 2 10.1186/1755-8166-4-2 21235775 PMC3033356

[B24] She X Horvath JE Jiang Z Liu G Furey TS Christ L Clark R Graves T Gulden CL Alkan C Bailey JA Sahinalp C Rocchi M Haussler D Wilson RK Miller W Schwartz S Eichler EE The structure and evolution of centromeric transition regions within the human genome Nature 2004 430 857 864 10.1038/nature02806 15318213

[B25] Lengauer C Green ED Cremer T Fluorescence in situ hybridization of YAC clones after Alu-PCR amplification Genomics 1992 13 826 828 10.1016/0888-7543(92)90160-T 1639408

[B26] Liehr T Heller A Starke H Claussen U FISH banding methods: applications in research and diagnostics Exp Rev Mol Diagn 2002 2 217 225 10.1586/14737159.2.3.217 12050860

[B27] Weise A Starke H Heller A Tönnies H Volleth M Stumm M Senger G Nietzel A Claussen U Liehr T Chromosome 2 aberrations in clinical cases characterised by high resolution multicolour banding and region specific FISH probes J Med Genet 2002 39 434 439 10.1136/jmg.39.6.434 12070255 PMC1735147

[B28] Manvelyan M Schreyer I Höls-Herpertz I Köhler S Niemann R Hehr U Belitz B Bartels I Götz J Huhle D Kossakiewicz M Tittelbach H Neubauer S Polityko A Mazauric ML Wegner R Stumm M Küpferling P Süss F Kunze H Weise A Liehr T Mrasek K Forty-eight new cases with infertility due to balanced chromosomal rearrangements: detailed molecular cytogenetic analysis of the 90 involved breakpoints Int J Mol Med 2007 19 855 864 17487417 10.3892/ijmm.19.6.855

[B29] Liehr T Karamysheva T Merkas M Brecevic L Hamid AB Ewers E Mrasek K Kosyakova N Weise A Somatic mosaicism in cases with small supernumerary marker chromosomes Curr Genomics 2010 11 432 439 10.2174/138920210793176029 21358988 PMC3018724

[B30] Weise A Mrasek K Fickelscher I Claussen U Cheung SW Cai WW Liehr T Kosyakova N Molecular definition of high-resolution multicolor banding probes: first within the human DNA sequence anchored FISH banding probe set J Histochem Cytochem 2008 56 487 493 10.1369/jhc.2008.950550 18256020 PMC2324187

[B31] Christian SL Fantes JA Mewborn SK Huang B Ledbetter DH Large genomic duplicons map to sites of instability in the prader-willi/angelman syndrome chromosome region (15q11-q13) Hum Mol Genet 1999 8 1025 1037 10.1093/hmg/8.6.1025 10332034

[B32] Torres-Juan L Rosell J Sanchez-de-la-Torre M Fibla J Heine-Suñer D Analysis of meiotic recombination in 22q11.2, a region that frequently undergoes deletions and duplications BMC Med Genet 2007 8 14 17397557 10.1186/1471-2350-8-14 PMC1855045

[B33] Li P Pomianowski P DiMaio MS Florio JR Rossi MR Xiang B Xu F Yang H Geng Q Xie J Mahoney MJ Genomic characterization of prenatally detected chromosomal structural abnormalities using oligonucleotide array comparative genomic hybridization Am J Med Genet A 2011 155 1605 1615 10.1002/ajmg.a.34043 21671377 PMC3745591

[B34] Slavin TP Kuruvilla K Curtis CA Christ LA Mitchell AL Isolated skeletal malformations in a child with a small mosaic ring microduplication of 18 p11.21q11.2: Genotype-phenotype correlations Am J Med Genet A 2011 155 618 621 10.1002/ajmg.a.33816 21344631 PMC4467729

[B35] Giardino D Bettio D Gottardi G Rizzi N Pierluigi M Perfumo C Calì A Dagna Bricarelli F Larizza L FISH characterization of two supernumerary r(1) associated with distinct clinical phenotypes Am J Med Genet 1999 84 3770 3780 10340656

[B36] Kosztolányi G Brecevic L Bajnòczky K Schinzel A Riegel M Mosaic supernumerary ring chromosome 1 in a three-generational family: 10-year follow-up report Eur J Med Genet 2011 54 152 156 10.1016/j.ejmg.2010.11.015 21145991

[B37] Liehr T Nietzel A Starke H Heller A Weise A Kuechler A Senger G Ebner S Martin T Stumm M Wegner R Tönnies H Hoppe C Claussen U Von Eggeling F Characterization of small marker chromosomes (SMC) by recently developed molecular cytogenetic approaches J Ass Genet Techn 2003 29 5 10 15213421

[B38] Tönnies H Hennies HC Spohr HL Neitzel H Characterization of the first supernumerary tricentric ring chromosome 1 mosaicism by conventional and molecular cytogenetic techniques Cytogenet Genome Res 2003 103 28 33 10.1159/000076285 15004460

[B39] Rodriguez L Starke H Guardia NM Tönnies H Neitzel H Kozlowski P Mazauric ML Heller A Grondona FL Mansilla E Santos Muñoz MJ Liehr T Martínez-Frías ML Three new cases with a supernumerary ring chromosome 1 Clin Dysmorphol 2005 14 169 175 10.1097/00019605-200510000-00001 16155417

[B40] Liehr T Stumm M Wegner RD Bhatt S Hickmann P Patsalis PC Meins M Morlot S Klaschka V Ewers E Hinreiner S Mrasek K Kosyakova N Cai WW Cheung SW Weise A 10p11.2 to 10q11.2 is a yet unreported region leading to unbalanced chromosomal abnormalities without phenotypic consequences Cytogenet Genome Res 2009 124 102 105 10.1159/000200094 19372675

[B41] Lichter P Tang Ghang CJ Call K Hermanson G Evans GA Housman D Ward DC High resolution mapping of human chromosome 11 by in situ hybridization with cosmid clones Science 1990 247 64 69 10.1126/science.2294592 2294592

[B42] Lichter P Cremer T Rooney DE, Czepulkowski BH Chromosome analysis by non-isotopic in situ hybridization Human cytogenetics. A practical approach 1992 Oxford: IRL Press at Oxford University Press 157 192

