# Supplementary material for: Design and validation of a pericentromeric BAC clone set aimed at improving diagnosis and phenotype prediction of supernumerary marker chromosomes
Source: Mol Cytogenet. 2013 Oct 30;6:45. doi: 10.1186/1755-8166-6-45 (PMC4176193; doi:10.1186/1755-8166-6-45)
Supplement: Additional file 1: Table S1 — Detailed list of the collected 486 pericentromeric BAC probes, including the 214 clones which belong to the high-resolution core panels, according to the UCSC Genome Browser Database assembly hg19. [file 1755-8166-6-45-S1.doc]

**Table S1 Detailed list of the collected 486 pericentromeric BAC probes, including the 214 clones which belong to the high-resolution core panels (in bold), according to the UCSC Genome Browser Database assembly hg19**

| **Chromosome**  **arms** | | **Core BAC clones** | **Chromosomal band** | **Physical position*^a^*** | **Cross-hybridizations*^b^*** | |
| --- | --- | --- | --- | --- | --- | --- |
|  | |  |  |  | Main | Minor |
|  | |  |  |  |  |  |
| **1p*^c^*** | | RP11-88D6 | 1p13-p12 | 117,710,721-117,919,282 |  |  |
|  |  | RP11-1069N17 | 1p12 | 117,856,141-118,039,786 |  |  |
|  |  | RP11-16G18 | 1p12 | 118,123,813-118,303,140 | + |  |
|  |  | RP11-134N8 | 1p12 | 118,201,801-118,352,585 |  |  |
|  |  | RP11-716A14 | 1p12 | 118,390,525-118,591,756 |  |  |
|  |  | RP11-806J7 | 1p12 | 118,567,596-118,777,756 |  |  |
|  |  | RP11-63I1 | 1p12 | 118,741,755-118,911,957 | + |  |
|  |  | RP11-828N6 | 1p12 | 118,991,214-119,207,287 | + |  |
|  |  | RP11-449E6 | 1p12 | 119,142,335-119,341,002 |  |  |
|  |  | RP11-50I4 | 1p12 | 119,269,705-119,471,574 |  |  |
|  |  | RP11-1073J15 | 1p12 | 119,421,980-119,603,572 |  |  |
|  |  | RP11-698L9 | 1p12 | 119,549,989-119,712,059 | + |  |
|  |  | RP11-1089J18 | 1p12 | 119,673,353-119,884,016 |  |  |
|  |  | RP11-46G23 | 1p12 | 119,852,664-120,024,783 |  |  |
|  |  | RP11-22F13 | 1p12 | 120,170,389-120,345,324 |  |  |
|  |  | RP11-320L8 | 1p12 |  | + |  |
|  |  | RP11-125P23 | 1p12 |  | + |  |
|  |  | **CTD-3138A9** | 1p12-p11.2 | 120,499,952-120,696,242 | 1q12-q21 | 15q26.3 |
|  |  | **RP11-671M21** | 1p11.2 | 120,747,163-120,898,555 | 1q12-q21, 1q32 |  |
|  |  | **RP11-206H22** | 1p11.2 | 121,157,866-121,312,250 |  | 4q11-q12 or 5q11.1-q11.2, 9q34.3 |
|  |  | **RP11-803J8** | 1p11.2 | 121,307,766-121,485,415 | 17p11.2-p11.1, 1912-q12 |  |
|  | |  |  |  |  |  |
| **1q*^c^*** | | **RP11-15M9** | 1q21.1 | 143,363,250-143,526,564 | 13p13, 13p11.2, 14p13, 14p11.2, 15p13, 15p11.2, 21p13, 21p11.2, 22p13, 22p11.2 | 4p12-p11 |
|  |  | **RP11-59H11** | 1q21.1 | 143,890,320-144,086,732 | 1p13-p12, 1q31-q32, 18q11.1-q11.2 | 17q11.1-q11.2 |
|  |  | **CTD-2326L14** | 1q21.1 | 144,507,758-144,822,788 | 1p13-p12 | 1p36.1 |
|  |  | RP11-248P4 | 1q21.1 | 144,506,978-144,845,907 | + |  |
|  |  | RP11-639G24 | 1q21.1 | 144,965,050-145,230,315 | + |  |
|  |  | RP11-164O19 | 1q21.1 | 145,211,878-145,374,409 | + |  |
|  |  | RP11-30I17 | 1q21.1 | 145,462,695-145,650,534 |  |  |
|  |  | RP11-257J15 | 1q21.1 | 149,084,929-149,260,214 |  |  |
|  |  | RP11-273D7 | 1q21.2 | 149,232,383-149,394,591 |  |  |
|  |  | RP11-160L8 | 1q21.2 | 149,271,969-149,435,668 |  |  |
|  |  | RP11-141M6 | 1q21.2 | 149,280,968-149,440,478 |  |  |
|  |  | RP11-65G1 | 1q21.2 | 149,552,493-149,716,359 |  |  |
|  | |  |  |  |  |  |
| **2p** | | RP11-495B16 | 2p12 | 82,758,168-82,862,954 |  |  |
|  |  | RP11-554H10 | 2p11.2 | 88,592,838-88,791,531 |  |  |
|  |  | RP11-1023A24 | 2p11.2 | 88,752,635-88,941,197 |  |  |
|  |  | RP11-260M7 | 2p11.2 | 88,874,772-89,043,151 |  |  |
|  |  | RP11-1084C10 | 2p11.2 | 89,045,768-89,270,254 | + |  |
|  |  | RP11-909L14 | 2p11.2 | 89,309,557-89,478,397 |  |  |
|  |  | RP11-1023H22 | 2p11.2 | 89,890,748-90,062,923 |  |  |
|  |  | RP11-3G5 | 2p11.2 | 89,924,516-90,080,121 |  |  |
| **Tab. S1 Continued** | | | | | | |
| **2p** | | **RP11-1023H22** | 2p11.2 | 89,890,748-90,062,923 |  |  |
|  |  | **RP11-97F19** | 2p11.2 | 90,021,054-90,195,695 | 2q37.3 |  |
|  |  | **RP11-433C18** | 2p11.2 | 90,163,552-90,321,509 | 1q12-q21.1, 15q11.2, 16p11.2-p11.1, 22q11.21 | 9p12-p11, 9q12-q13, 14p11.2, 15p11.2 |
|  |  | **CTD-2269O20** | 2p11.2-p11.1 | 90,419,429-90,500,020 | 1q12-q21.1, 2p14-p13, 3p12-p11 | 7p12-p11, 9p12-p11, 9q12-q13, 14p11.2, 15q11.2, 22q11.21 |
|  |  | RP11-451G6 | 2p11.1 | 91,599,417-91,728,215 | + |  |
|  |  | CTD-2509A14 | 2p11.1 | 91,692,777-91,839,948 | + |  |
|  |  | RP11-165D20 | 2p11.1 | 91,831,913-91,989,954 | + |  |
|  |  | RP11-1069D4 | 2p11.1 | 92,051,347-92,266,848 | + |  |
|  |  | CTD-2182l24 | 2p11.1 | 92,214,111-92,303,664 | + |  |
|  |  | RP11-469P16 | 2p11.1 | 92,273,168-92,319,515 | + |  |
|  | |  |  |  |  |  |
| **2q** | | RP11-134N21 | 2q11.1 | 95,326,172-95,495,021 | + |  |
|  |  | RP11-71B7 | 2q11.1 | 95,418,410-95,619,971 | + |  |
|  |  | RP11-708D7 | 2q11.1 | 95,617,775-95,787,635 |  |  |
|  |  | CTD-2367P13 | 2q11.1 | 95,862,699-95,976,987 |  |  |
|  |  | RP11-468G5 | 2q11.1 | 95,903,645-96,106,005 |  |  |
|  |  | RP11-345I9 | 2q11.1 | 95,913,888-96,085,700 |  |  |
|  |  | RP11-188C16 | 2q11.1 | 96,096,206-96,255,712 | + |  |
|  |  | RP11-351J10 | 2q11.1 | 96,244,657-96,411,310 | + |  |
|  |  | RP11-34G16 | 2q11.1 | 96,312,034-96,499,916 | + |  |
|  |  | **RP11-257B11** | 2q11.1 | 96,499,808-96,658,815 | 9p11.2, 15p11.2 |  |
|  |  | **RP11-139J5** | 2q11.1-q11.2 | 96,658,860-96,836,396 |  |  |
|  |  | **RP11-574O17** | 2q11.2 | 96,787,401-96,933,318 |  |  |
|  |  | **RP11-799H7** | 2q11.2 | 96,929,975-97,134,699 |  |  |
|  |  | **RP11-72L20** | 2q11.2 | 97,099,682-97,252,549 | 1q25 |  |
|  |  | **RP11-466M9** | 2q11.2 | 97,240,932-97,412,500 |  |  |
|  |  | **RP11-245P4** | 2q11.2 | 97,591,299-97,764,909 |  |  |
|  |  | RP11-1069F6 | 2q11.2 | 97,731,040-97,939,773 |  |  |
|  |  | RP11-1082A11 | 2q11.2 | 97,921,167-98,099,469 |  |  |
|  |  | RP11-716G6 | 2q11.2 | 98,265,109-98,446,561 |  |  |
|  |  | RP11-973B20 | 2q11.2 | 98,363,671-98,535,867 | + |  |
|  |  | RP11-947L13 | 2q11.2 | 98,514,812-98,699,095 |  |  |
|  |  | RP11-1020K12 | 2q11.2 | 98,670,398-98,860,435 |  |  |
|  |  | RP11-411B19 | 2q11.2 | 98,832,869-99,000,049 |  |  |
|  |  | RP11-321A19 | 2q11.2 | 100,638,560-100,836,578 |  |  |
|  | |  |  |  |  |  |
| **3p** | | RP11-81D17 | 3p12.2-p12.1 | 83,371,622-83,523,402 | + |  |
|  |  | RP11-79F5 | 3p12.1 | 83,886,473-84,069,201 | + |  |
|  |  | RP11-655A17 | 3p12.1 | 87,017,008-87,187,800 | + |  |
|  |  | **RP11-598A10** | 3p12.1-p11.2 | 87,186,532-87,387,150 |  |  |
|  |  | **RP11-905J12** | 3p11.2 | 87,383,920-87,584,086 | 3p26-p25, 5q31, 7q11.2, 18q23 | 2p12-p11.2, 2q32, 12q24.33, 17q21 |
|  |  | **RP11-1082I19** | 3p11.2 | 87,569,564-87,784,200 | 16p13.3, 19p13.3 | 6q24q25, 11p15.5, 11q22-q23, 18q11.2-q12, 18q23 |
|  |  | **RP11-424C9** | 3p11.2-p11.1 | 87,830,799-87,996,255 | 11p15.5 | 1q24-q25 |
|  |  | **RP11-159G9** | 3p11.1 | 88,035,949-88,190,732 |  |  |
|  |  | **RP11-591J20** | 3p11.1 | 88,122,058-88,268,542 | 10p14-p13 |  |
|  |  | RP11-135M3 | 3p11.1 | 88,846,738-89,029,254 |  |  |
|  |  | RP11-164C5 | 3p11.1 | 88,961,650-89,134,538 |  |  |
| **Tab. S1 Continued** | | | | | | |
| **3p** | | RP11-805E22 | 3p11.1 | 89,083,909-89,286,888 |  |  |
|  |  | RP11-47O5 | 3p11.1 | 89,354,276-89,528,041 |  |  |
|  |  | CTD-3063D5 | 3p11.1 | 89,532,916-89,715,374 |  |  |
|  |  | RP11-655P10 | 3p11.1 | 89,682,175-89,853,866 | + |  |
|  | |  |  |  |  |  |
| **3q** | | RP11-124L3 | 3q11.1 | 93,504,855-93,629,605 | + |  |
|  |  | **CTD-3154B7** | 3q11.1 | 93,591,223-93,720,390 |  |  |
|  |  | **RP11-13K6** | 3q11.1 | 93,683,242-93,859,578 |  |  |
|  |  | **RP11-259L20** | 3q11.1- q11.2 | 93,796,399-93,956,627 |  | 15q22 |
|  |  | **RP11-1133P14** | 3q11.2 | 93,917,956-94,088,792 |  |  |
|  |  | **RP11-1069E2** | 3q11.2 | 94,399,115-94,571,096 |  |  |
|  |  | **RP11-625F19** | 3q11.2 | 96,619,911-94,771,613 |  |  |
|  |  | RP11-734G18 | 3q11.2 | 94,702,775-94,884,608 | + |  |
|  |  | RP11-91M15 | 3q11.2 | 94,960,454-95,145,238 | + |  |
|  |  | RP11-449F7 | 3q11.2 | 97,566,313-97,775,848 |  |  |
|  | |  |  |  |  |  |
| **4p** | | RP11-178N2 | 4p12 | 45,736,237-45,902,428 |  |  |
|  |  | RP11-90F11 | 4p12 | 46,147,638-46,303,811 |  |  |
|  |  | **CTD-2057N12** | 4p12 | 47,320,259-47,494,227 |  |  |
|  |  | **CTD-2199P1** | 4p12 | 47,601,553-47,832,534 |  |  |
|  |  | **RP11-121C2** | 4p12 | 47,803,222-48,024,636 | 10q24-q25 |  |
|  |  | **RP11-260K18** | 4p12-p11 | 48,093,213-48,252,013 |  |  |
|  |  | **RP11-809H21** | 4p11 | 48,252,022-48,467,680 |  |  |
|  |  | **RP11-665L9** | 4p11 | 48,523,264-48,691,337 |  |  |
|  |  | RP11-192F3 | 4p11 | 48,721,320-48,875,593 |  |  |
|  |  | CTD-3149N21 | 4p11 | 48,825,996-49,065,590 |  |  |
|  |  | RP11-620D19 | 4p11 | 49,065,609-49,246,655 | + |  |
|  |  | RP11-351N21 | 4p11 | 49,199,285-49,541,358 and  49,258,015- 49,601,461 | + |  |
|  |  | RP11-1122F17 | 4p11 | 49,516,051-49,598,500  and 49,516,051-49,595,687 | + |  |
|  | |  |  |  |  |  |
| **4q** | | **RP11-98B6** | 4q11-q12 | 52,681,899-52,856,481 |  |  |
|  |  | **RP11-420L16** | 4q12 | 52,875,265-53,027,277 |  |  |
|  |  | **RP11-796H19** | 4q12 | 52,989,056-53,165,523 |  |  |
|  |  | **RP11-600M5** | 4q12 | 53,277,748-53,456,236 |  |  |
|  |  | RP11-89B16 | 4q12 | 54,644,312-54,799,635 |  |  |
|  |  | RP11-345F18 | 4q12 | 56,651,921-56,847,075 |  |  |
|  | |  |  |  |  |  |
| **5p** | | RP11-170D20 | 5p12 | 42,514,255-42,688,013 |  |  |
|  |  | RP11-433J10 | 5p12 | 43,065,762-43,251,626 |  |  |
|  |  | RP11-197M6 | 5p12 | 44,053,543-44,209,628 |  |  |
|  |  | RP11-451A9 | 5p12 | 44,443,972-44,636,101 |  |  |
|  |  | **RP11-134N5** | 5p12 | 45,188,195-45,357,985 |  |  |
|  |  | **RP11-929P16** | 5p12 | 45,300,254-45,510,055 |  |  |
|  |  | **RP11-1110N13** | 5p12 | 45,414,584-45,586,548 |  |  |
|  |  | **RP11-624L15** | 5p12 | 45,645,984-45,850,256 |  |  |
|  |  | **RP11-996C23** | 5p12 | 45,804,705-45,985,047 |  |  |
|  |  | **RP11-10F16** | 5p12-p11 | 45,985,058-46,179,715 |  |  |
|  | |  |  |  |  |  |
|  | |  |  |  |  |  |
|  | |  |  |  |  |  |
| **Tab. S1 Continued** | | | | | | |
| **5q** | | RP11-366L3 | 5q11.1 | 49,574,635-49,767,536 | + |  |
|  |  | RP11-124E22 | 5q11.1 | 49,886,693-50,059,738 | + |  |
|  |  | RP11-91I22 | 5q11.1 | 50,072,147-50,238,865 |  |  |
|  |  | **RP11-109H24** | 5q11.1 | 50,403,599-50,568,595 | 1q42 |  |
|  |  | **RP11-242H13** | 5q11.1 | 50,480,654-50,663,488 |  |  |
|  |  | **RP11-463E10** | 5q11.1-q11.2 | 50,651,421-50,835,017 |  |  |
|  |  | **CTD-3066J14** | 5q11.2 | 50,803,458-50,975,114 | 3p21, 9q13 | 4q21 |
|  |  | **CTD-3113M11** | 5q11.2 | 50,888,975-51,035,561 |  |  |
|  |  | RP11-780G5 | 5q11.2 | 52,410,255-52,618,788 | + |  |
|  |  | RP11-332C17 | 5q11.2 | 55,030,386-55,236,842 |  |  |
|  |  | RP11-143K14 | 5q11.2 | 55,683,172-55,868,279 |  |  |
|  | |  |  |  |  |  |
| **6p** | | RP11-111P16 | 6p12.1 | 55,436,218-55,607,837 |  |  |
|  |  | RP11-79O24 | 6p12.1 | 56,100,823-56,273,125 |  |  |
|  |  | RP11-519E3 | 6p12.1-p11.2 | 56,896,846-57,081,575 |  |  |
|  |  | RP11-313P7 | 6p11.2 | 57,131,187-57,315,164 |  |  |
|  |  | **RP11-799H20** | 6p11.2 | 57,679,122-57,892,749 | 6q11-q12 |  |
|  |  | **RP11-452D24** | 6p11.2 | 57,921,142-58,087,659 |  |  |
|  |  | **RP11-357F21** | 6p11.2 | 58,137,746-58,269,362 | 5p15.1-p14, 5p13-p12, 5q13, 6p22-p21 | 5q14-q21 |
|  |  | **RP11-436E23** | 6p11.2 | 58,295,594-58,463,021 | 6q11 |  |
|  |  | **RP11-136G2** | 6p11.2-p11.1 | 58,612,630-58,780,147 |  |  |
|  | |  |  |  |  |  |
| **6q** | | RP11-164C22 | 6q11.1 | 62,321,506-62,519,239 |  |  |
|  |  | RP11-878F11 | 6q11.1 | 62,512,059-62,685,178 | + |  |
|  |  | RP11-120I19 | 6q11.1 | 62,622,405-62,792,860 | + |  |
|  |  | **RP11-1077J15** | 6q11.1 | 62,806,182-62,992,837 |  |  |
|  |  | **CTD-2057E3** | 6q11.1 | 63,000,211-63,156,755 |  |  |
|  |  | **RP11-448N11** | 6q11.1-q11.2 | 63,149,602-63,353,102 |  |  |
|  |  | **RP11-313N17** | 6q11.2-q12 | 63,293,649-63,451,135 |  |  |
|  |  | **RP11-78B14** | 6q12 | 63,435,128-63,601,366 | 10q25.3 |  |
|  |  | RP11-79F21 | 6q12 | 64,254,154-64,428,051 | + |  |
|  |  | RP11-220P17 | 6q12 | 65,943,195-66,116,974 | + |  |
|  |  | RP11-502L6 | 6q12 | 69,675,167-69,850,722 |  |  |
|  | |  |  |  |  |  |
| **7p** | | RP11-90N11 | 7p12.1-p11.2 | 53,930,517-54,090,412 | + |  |
|  |  | RP11-89E8 | 7p11.2 | 55,114,053-55,294,787 | + |  |
|  |  | RP11-80L24 | 7p11.2 | 55,876,172-56,010,309 | + |  |
|  |  | RP11-15K19 | 7p11.2 | 56,007,288-56,127,272 |  |  |
|  |  | RP11-409A23 | 7p11.2 | 56,106,883-56,256,101 |  |  |
|  |  | RP11-1107C11 | 7p11.2 | 56,534,796-56,731,394 | + |  |
|  |  | RP11-1105M8 | 7p11.2 | 56,783,163-56,925,306 | + |  |
|  |  | RP11-25D18 | 7p11.2 | 57,159,724-57,325,034 | + |  |
|  |  | **RP11-114G11*^d^*** | 7p11.2 | 57,342,227-57,520,331 |  |  |
|  |  | **RP11-736D24** | 7p11.2 | 57,529,035-57,718,892 | 20p11.2 | 13p11.2; 14p11.2; 15p11.2; 17p11.2; 21p11.2; 22p11.2 |
|  |  | **RP11-357N20** | 7p11.2 | 57,802,218-57,976,763 | 17p11.2; 20p11.2 | 9p12; 9q12; 13p11.2; 14p11.2; 15p11.2; 16p11.2; 20q11.2; 21p11.2; 22p11.2 |
|  |  | **CTD-2593N8** | 7p11.2-p11.1 | 57,898,343-58,054,325 | 9p12, 13p11.2-p11.1, 14p11.2-p11.1, 15q11.1-q11.2, 22p11.2-p11.1 | 17p11.2-p11.1, 21q11.1-q11.2 |
|  | |  |  |  |  |  |
|  | |  |  |  |  |  |
| **Tab. S1 Continued** | |  |  |  |  |  |
| **7q** | | RP11-432A1 | 7q11.1 | 61,083,478-61,276,580 |  |  |
|  |  | **CTD-2245O1** | 7q11.1-q11.21 | 61,573,732-61,819,063 |  |  |
|  |  | **CTD-2116L11** | 7q11.21 | 61,767,899-61,884,234 |  |  |
|  |  | **CTD-3154D5** | 7q11.21 | 61,980,918-62,134,464 | 1q12 |  |
|  |  | **RP11-1134G5** | 7q11.21 | 62,150,710-62,290,949 |  |  |
|  |  | **RP11-45N18** | 7q11.21 | 62,377,875-62,539,803 |  |  |
|  |  | RP11-90C3 | 7q11.21 | 64,670,960-64,830,210 |  |  |
|  |  | RP11-118D11 | 7q11.21-q22 | 66,878,316-67,036,341 |  |  |
|  | |  |  |  |  |  |
| **8p** | | RP11-133O7 | 8p11.21 | 39,837,737-39,838,225 |  |  |
|  |  | RP11-282C12 | 8p11.21 | 41,980,132-42,182,818 |  |  |
|  |  | RP11-959A8 | 8p11.21 | 42,150,637-42,326,388 |  |  |
|  |  | **RP11-577C12** | 8p11.21 | 42,310,344-42,524,015 |  |  |
|  |  | **RP11-89A4** | 8p11.21 | 42,577,154-42,731,336 |  |  |
|  |  | **RP11-73M19** | 8p11.21-p11.1 | 42,983,674-43,151,133 |  |  |
|  |  | **RP11-195E9** | 8p11.1 | 43,287,371-43,459,059 |  |  |
|  |  | **RP11-643N23** | 8p11.1 | 43,419,504-43,605,038 |  |  |
|  | |  |  |  |  |  |
| **8q** | | RP11-747K13 | 8q11.1 | 47,080,192-47,240,641 |  |  |
|  |  | RP11-3N5 | 8q11.1 | 47,221,552-47,380,415 |  |  |
|  |  | RP11-17A22 | 8q11.1 | 47,336,605-47,509,000 |  |  |
|  |  | **RP11-799D18** | 8q11.1 | 47,474,079-47,667,207 |  |  |
|  |  | **RP11-153L10** | 8q11.1 | 47,609,322-47,781,223 | Yp11.2-p11.1 |  |
|  |  | **RP11-1083M7** | 8q11.1 | 47,747,827-47,955,319 |  |  |
|  |  | **CTD-2563N10** | 8q11.1-q11.21 | 47,961,101-48,172,122 |  |  |
|  |  | **RP11-933K15** | 8q11.21 | 48,308,268-48,524,278 |  |  |
|  |  | **RP11-367A12** | 8q11.21 | 48,487,561-48,660,399 |  |  |
|  |  | RP11-13I4 | 8q11.21 | 48,623,678-48,800,587 |  |  |
|  |  | RP11-959L20 | 8q11.21 | 48,788,451-48,966,769 |  |  |
|  |  | RP11-68L8 | 8q11.21 | 48,966,770-49,138,034 |  |  |
|  |  | RP11-268N2 | 8q11.21 | 49,067,789-49,068,275 |  |  |
|  |  | RP11-123I15 | 8q11.21 | 49,112,699-49,307,732 |  |  |
|  |  | RP11-951D12 | 8q11.21 | 49,279,311-49,477,842 |  |  |
|  | |  |  |  |  |  |
| **9p*^c^*** | | RP11-135J15 | 9p12 | 40,508,574-40,660,148 | + |  |
|  |  | RP11-133G22 | 9p12 | 40,513,339-40,659,287 | + |  |
|  |  | RP11-86I14 | 9p12 | 42,208,320-42,380,036 |  |  |
|  |  | RP11-115C9 | 9p11.2 | 43,996,577-44,146,438 | + |  |
|  |  | RP11-14P3 | 9p11.2 | 44,366,120-44,510,065 | + |  |
|  |  | **RP11-361G22** | 9p11.2 | 46,287,332-46,461,039 | 9q13, 12q13-q14, 20p11.2-p11.1, 20q11.1-q11.2 | 4q31, 13p11.2-p11.1, 14p11.2-p11.1, 15p11.2-p11.1, 17p11.2, 21p11.2-p11.1, 22p11.2-p11.1 |
|  |  | **RP11-977H12** | 9p11.2 | 46,617,899-46,784,244 | 9q12-q13 |  |
|  |  | **RP11-606B18** | 9p11.2 | 46,676,056-46,926,948 | 9q12-q13 |  |
|  | |  |  |  |  |  |
| **9q** | | **RP11-795P22** | 9q12 | 65,480,845-65,688,126 | 9p13-p12 |  |
|  |  | **CTD-2508M5** | 9q12-q13 | 65,752,712-65,918,354 | 9p13.1 |  |
|  |  | **RP11-348I24** | 9q13 | 65,978,548-66,154,396 | 9p13-p12 |  |
|  |  | **CTD-2050L17** | 9q13 | 66,246,709-66,391,027 | 2p11.2, 9p13-p12 | 14q11.2, 21q11.1-q11.2, 22p11.2 |
|  |  | RP11-76I5 | 9q13 | 67,631,906-67,784,266 |  |  |
|  |  | RP11-203G10 | 9q21.11 | 69,533,757-69,708,240 | + |  |
| **Tab. S1 Continued** | | | | | | |
| **9q** | | RP11-35I5 | 9q21.11 | 70,391,073-70,506,535 | + |  |
|  |  | RP11-154P18 | 9q21.11 | 70,556,542-70,735,467 | + |  |
|  |  | RP11-151I14 | 9q21.11 | 70,850,699-71,031,447 | + |  |
|  |  | RP11-76I20 | 9q21.11 | 70,996,539-71,177,602 |  |  |
|  |  | RP11-474L15 | 9q21.11 | 71,152,068-71,314,595 |  |  |
|  |  | RP11-203L2 | 9q21.11 | 71,258,100-71,452,811 | + |  |
|  | |  |  |  |  |  |
| **10p** | | RP11-134A8 | 10p11.21 | 35,400,139-35,519,878 |  |  |
|  |  | **RP11-739D18** | 10p11.21 | 37,422,279-37,507,938 |  |  |
|  |  | **RP11-20F24** | 10p11.21 | 37,505,939-37,660,103 | 1p36.1 |  |
|  |  | **RP11-420N14** | 10p11.21 | 37,684,896-37,856,050 |  |  |
|  |  | **CTD-3195G22** | 10p11.21-p11.1 | 37,913,276-38,091,971 |  |  |
|  |  | **RP11-113O13** | 10p11.1 | 38,258,780-38,402,598 |  |  |
|  |  | **RP11-133H16** | 10p11.1 | 38,402,605-38,579,714 | 10q11.2 | 1q43 |
|  |  | CTD-2530C6 | 10p11.1 | 38,473,991-38,701,167 |  |  |
|  |  | RP11-291L22 | 10p11.1 | 38,652,001-38,818,835 | + |  |
|  |  | CTD-2281G15 | 10p11.1 | 38,958,780-39,047,292 | + |  |
|  | |  |  |  |  |  |
| **10q*^c^*** | | **RP11-80L2** | 10q11.21 | 42,383,288-42,396,242 |  |  |
|  |  | **RP11-178A10** | 10q11.21 | 42,877,687-43,000,769 |  |  |
|  |  | **RP11-351D16** | 10q11.21 | 43,497,191-43,702,986 | 1q12 |  |
|  |  | RP11-89J23 | 10q11.21 | 44,112,320-44,276,126 |  |  |
|  |  | RP11-172C24 | 10q11.21 | 45,883,489-46,043,203 |  |  |
|  | |  |  |  |  |  |
| **11p*^c^*** | | RP11-12D19 | 11p12 | 43,725,092-43,886,180 | + |  |
|  |  | RP11-79A4 | 11p12-p11.12 | 48,687,899-48,844,595 |  |  |
|  |  | RP11- 15F22 | 11p11.12 | 49,034,936-49,185,191 | + |  |
|  |  | RP11-1062A8 | 11p11.12 | 49,052,012-49,259,387 | + |  |
|  |  | RP11-746P9 | 11p11.12 | 50,111,641-50,289,199 |  |  |
|  |  | RP11-806K4 | 11p11.12 | 50,285,491-50,470,497 |  |  |
|  |  | **RP11-318O24** | 11p11.12 | 50,589,277-50,763,373 |  |  |
|  |  | **RP11-685O18** | 11p11.12 | 51,137,798-51,337,514 |  |  |
|  |  | **RP11-296O21** | 11p11.12 | 51,287,414-51,475,639 | 11p11.1-q11 |  |
|  |  | **RP11- 100E23** | 11p11.12 | 51,438,409-51,594,205 | 11p11.1-q11 |  |
|  | |  |  |  |  |  |
| **11q** | | RP11-79G14 | 11q11 | 54,728,719-54,971,384 | + |  |
|  |  | RP11-813P9 | 11q11 | 54,955,503-55,158,099 | + |  |
|  |  | **RP11-241O17** | 11q11 | 55,429,655-55,594,445 |  |  |
|  |  | **CTD-3202L3** | 11q11-q12.1 | 55,628,589-55,767,588 | 11p11.2-p11.1 |  |
|  |  | **CTD-2526A11** | 11q12.1 | 55,746,871-55,948,568 |  |  |
|  |  | **RP11-1095H10** | 11q12.1 | 55,829,634-56,018,373 | 3q27-q29, 20q12-q13.1 | 2p16-p13, 8q22-q23, 13q32-q33, 15q21-q22 |
|  |  | **RP11-85E22** | 11q12.1 | 55,914,685-56,080,521 |  |  |
|  |  | **RP11-592B9** | 11q12.1 | 56,215,308-56,411,365 | 16q13-q21 |  |
|  |  | **RP11-720L5** | 11q12.1 | 56,392,688-56,551,742 | 11q22 |  |
|  |  | RP11-236L12 | 11q12.1 | 56,547,941-56,695,136 |  |  |
|  |  | RP11-163E6 | 11q12.1 | 56,705,651-56,876,653 |  |  |
|  |  | RP11-352C11 | 11q12.1 | 56,869,229-57,068,737 |  |  |
|  |  | RP11-872D17 | 11q12.1 | 57,012,158-57,202,408 |  |  |
|  | |  |  |  |  |  |
|  | |  |  |  |  |  |
| **Tab. S1 Continued** | |  |  |  |  |  |
| **11q** | | RP11-118N5 | 11q12.1 | 57,966,740-58,155,914 |  |  |
|  |  | RP11-168D22 | 11q12.2 | 60,165,532-60,342,322 | + |  |
|  |  | RP11-5F17 | 11q12.2 | 61,330,635-61,540,026 |  |  |
|  | |  |  |  |  |  |
| **12p** | | RP11-90H7 | 12p11.22-p11.21 | 30,570,587-30,749,400 |  |  |
|  |  | RP11-517B23 | 12p11.21 | 31,471,644-32,029,051 |  |  |
|  |  | RP11-372M9 | 12p11.21 | 31,981,789-32,172,587 |  |  |
|  |  | **RP11-8P13*^c^*** | 12p11.21 | 32,817,080-33,045,536 |  |  |
|  |  | **RP11-91O8** | 12p11.21 | 33,022,758-33,184,817 |  |  |
|  |  | **RP11-792O21** | 12p11.21 | 33,149,755-33,355,434 |  |  |
|  |  | **RP11-460N10** | 12p11.21-p11.1 | 33,279,249-33,442,226 |  |  |
|  |  | **RP11-974O7** | 12p11.1 | 33,368,133-33,528,720 | 13q21 |  |
|  |  | **RP11-88P4** | 12p11.1 | 33,468,677-33,643,594 |  | Xp22.3 |
|  |  | RP11-747M3 | 12p11.1 | 34,055,842-34,218,122 |  |  |
|  | |  |  |  |  |  |
| **12q** | | **RP11-95O1** | 12q11 | 38,020,543-38,185,706 |  | 13p11.2-p11.1, 14p11.2-p11.1, 15p11.2-p11.1, 21p11.2-p11.1, 22p11.2-p11.1, Yp11.2 |
|  |  | **RP11-496H24** | 12q11-12 | 38,115,907-38,275,937 |  |  |
|  |  | **RP11-164G9** | 12q12 | 38,291,466-38,463,095 |  |  |
|  |  | **RP11-266O15** | 12q12 | 38,574,199-38,741,450 |  |  |
|  |  | **RP11-715M8** | 12q12 | 38,714,300-38,875,078 |  |  |
|  |  | RP11-498B21 | 12q12 | 41,467,007-41,650,908 |  |  |
|  |  | RP11-463G20 | 12q12 | 45,419,834-45,619,603 | + |  |
|  | |  |  |  |  |  |
| **13q** | | **RP11-79H3** | 13q11 | 19,108,604-19,276,373 | 18p11.2-p11.1 | 21q11.1-q11.2 |
|  |  | **CTD-3188C8** | 13q11 | 19,273,648-19,456,931 | 2q11.1-q11.2, 3p25, 4p13-p11, 9p12-p11, 9q12-q13, 12p11.2, 18p11.2-p11.1, 21q11.1-q11.2 | 1p11-q11, 2q21, 3q11.1-q11.2, 9p13, 13p13, 13p11.2, 14p13, 14p11.2, 15p11.2, 16q11.2-q12, 21p13, 21p11.2, 22p13, 22p11.2 |
|  |  | **RP11-294G16** | 13q11-q12.11 | 19,408,142-19,561,335 |  | 21q11.1-q11.2 |
|  |  | **RP11-301J16** | 13q12.11 | 19,550,674-19,702,992 |  |  |
|  |  | **RP11-71I1** | 13q12.11 | 19,642,936-19,820,541 |  |  |
|  |  | RP11-61K9 | 13q12.11 | 20,323,995-20,488,914 |  |  |
|  |  | RP11-69J15 | 13q12.11 | 20,835,872-21,007,650 |  |  |
|  |  | RP11-45A5 | 13q12.11 | 21,526,704-21,680,318 |  |  |
|  |  | RP11-103L7 | 13q12.11 | 21,907,874-22,084,995 |  |  |
|  |  | RP11-101D3 | 13q12.11 | 22,437,523-22,601,807 |  |  |
|  |  | RP11-98L10 | 13q12.11 | 23,079,496-23,233,485 |  |  |
|  | |  |  |  |  |  |
| **14q** | | **RP11-639F13** | 14q11.1-q11.2 | 19,043,655-19,241,263 | 2q21-q22, 9p13-p12, 9q12-q13, 13q11-q12, 15q11.1-q11.2, 18p11.2, 21q11.1-q11.2, 22q11.1-q11.2 | 1p36.2-p36.1, 8q24.2-q24.3, 11p15-p14 |
|  |  | **RP11-134C5** | 14q11.2 | 19,307,335-19,454,103 | 2q21-q22, 18p11.2, 22q11.1-q11.2 | 13q11-q12, 15q11.1-q11.2, 21q11.1-q11.2 |
|  |  | **RP11-1112K21** | 14q11.2 | 19,465,071-19,607,628 | 2q21-q22, 22q11.1-q11.2 |  |
|  |  | **RP11-829D17** | 14q11.2 | 19,573,554-19,752,283 | 2q21-q22, 22q11.1-q11.2 | 15q11.1-q11.2 |
|  |  | **RP11-77E23** | 14q11.2 | 19,819,166-20,000,086 | 2q21-q22, 21q11.1-q11.2, 22q11.1-q11.2 | 15q11.1-q11.2 |
|  |  | RP11-354F21 | 14q11.2 | 20,000,085-20,192,582 | + |  |
|  |  | RP11-831B15 | 14q11.2 | 20,203,849-20,415,579 | + |  |
|  |  | RP11-14J7 | 14q11.2 | 20,940,682-21,103,092 |  |  |
|  |  | RP11-81F9 | 14q11.2 | 23,662,649-23,823,282 |  |  |
|  | |  |  |  |  |  |
|  | |  |  |  |  |  |
|  | |  |  |  |  |  |
| **Tab. S1 Continued** | |  |  |  |  |  |
| **15q** | | RP11-354I16 | 15q11.1 | 20,025,938-20,213,357 | + |  |
|  |  | RP11-936K13 | 15q11.1 | 20,186,010-20,356,444 | + |  |
|  |  | RP11-837N18 | 15q11.1 | 20,287,195-20,436,664 | + |  |
|  |  | **RP11-357P4** | 15q11.1 | 20,414,538-20,611,321 | 16p11.2 | 2p11.2 |
|  |  | **RP11-108C1** | 15q11.1-q11.2 | 20,578,131-20,754,085 |  |  |
|  |  | **RP11-779L1** | 15q11.2 | 20,675,460-20,850,175 |  |  |
|  |  | **RP11-349A12** | 15q11.2 | 20,790,489-20,997,363 | 22p11.2, 22q11.2 |  |
|  |  | **RP11-666L22** | 15q11.2 | 20,847,447-21,040,492 | 14q11.2, 18q11.1q11.2 |  |
|  |  | RP11-415B7 | 15q11.2 | 22,179,698-22,413,497 |  |  |
|  |  | RP11-816O23 | 15q11.2 | 22,370,184-22,572,123 |  |  |
|  |  | RP11-467N20 | 15q11.2 | 22,646,319-22,830,873 |  |  |
|  |  | RP11-307C10 | 15q11.2 | 22,973,229-23,141,039 |  |  |
|  |  | RP11-80H14 | 15q11.2 | 22,984,975-22,985,532 |  |  |
|  |  | RP11-75A6 | 15q11.2 | 23,309,953-23,475,913 | + |  |
|  |  | CTD-3190E6 | 15q11.2 | 23,437,164-23,720,453 |  |  |
|  |  | RP11-262J5 | 15q11.2 | 23,564,866-23,739,032 |  |  |
|  |  | RP11-262I11 | 15q11.2 | 23,661,494-23,814,757 | + |  |
|  |  | RP11-196F4 | 15q11.2 | 23,678,416-23,830,836 | + |  |
|  |  | RP11-373J1 | 15q11.2 | 23,887,760-24,087,140 |  |  |
|  |  | RP11-484P15 | 15q11.2 | 24,078,099-24,284,047 |  |  |
|  |  | RP11-243I19 | 15q11.2 | 24,314,154-24,629,020 |  |  |
|  |  | RP11-769E20 | 15q11.2 | 24,744,643-24,910,954 |  |  |
|  |  | RP11-662O22 | 15q11.2 | 24,952,713-25,122,373 | + |  |
|  |  | RP11-125E1 | 15q11.2 | 25,026,058-25,200,751 |  |  |
|  |  | RP11-94G4 | 15q11.2 | 25,098,201-25,270,989 |  |  |
|  |  | RP11-171C8 | 15q11.2 | 25,356,958-25,517,707 |  |  |
|  | |  |  |  |  |  |
| **16p** | | RP11-18H23 | 16p11.2 | 31,535,991-31,536,194 |  |  |
|  |  | RP11-431E24 | 16p11.2 | 31,741,538-31,927,175 |  |  |
|  |  | RP11-62P21 | 16p11.2 | 32,272,623-32,437,717 |  |  |
|  |  | RP11-264M14 | 16p11.2 | 33,374,950-33,545,220 | + |  |
|  |  | **RP11-1088B6** | 16p11.2 | 34,279,064-34,449,270 |  |  |
|  |  | **RP11-488I20** | 16p11.2-p11.1 | 34,431,661-34,632,711 |  |  |
|  |  | **RP11-80F22*^d^*** | 16p11.1 | 34,618,594-34,769,642 |  |  |
|  |  | **RP11-143K2** | 16p11.1 | 34,815,121-34,996,313 |  |  |
|  |  | **RP11-765F22** | 16p11.1 | 35,000,188-35,172,343 |  |  |
|  | |  |  |  |  |  |
| **16q** | | **RP11-91A22** | 16q11.2 | 46,520,735-46,692,088 |  |  |
|  |  | **CTD-2382P11** | 16q11.2 | 46,638,723-46,840,665 |  |  |
|  |  | **RP11-627O2** | 16q11.2-q12.1 | 46,843,904-47,021,633 |  |  |
|  |  | **RP11-283C7** | 16q11.2-q12.1 | 46,945,331-47,130,611 |  |  |
|  |  | **RP11-671L23** | 16q12.1 | 47,187,522-47,363,142 |  |  |
|  |  | RP11-474B12 | 16q12.1 | 47,316,866-47,469,918 |  |  |
|  | |  |  |  |  |  |
| **17p** | | RP11-31D8 | 17p12-p11.2 | 15,874,670-16,026,171 |  |  |
|  |  | RP11-404D6 | 17p11.2 | 16,265,217-16,455,279 | + |  |
|  |  | RP11-31D8 | 17p12-p11.2 | 15,874,670-16,026,171 |  |  |
|  | |  |  |  |  |  |
|  | |  |  |  |  |  |
| **Tab. S1 Continued** | |  |  |  |  |  |
| **17p** | | RP11-64J19 | 17p11.2 | 21,073,930-21,250,955 |  |  |
|  |  | RP11-45G12 | 17p11.2 | 21,250,948-21,400,203 |  |  |
|  |  | RP11-822E23 | 17p11.2 | 21,320,482-21,529,633 |  |  |
|  |  | **RP11-937K3** | 17p11.2 | 21,686,556-21,879,423 |  |  |
|  |  | **RP11-806L9** | 17p11.2 | 21,779,243-21,933,929 | 9p13, 9p12, 20p11.2 | 7p12-p11, 9q12-q13, 12p11.2-p11.1 |
|  |  | **CTD-2024B23** | 17p11.2 | 21,921,407-22,095,554 |  |  |
|  |  | **RP11-718K3** | 17p11.2-p11.1 | 22,122,006-22,263,000 |  |  |
|  | |  |  |  |  |  |
| **17q** | | RP11-25J2 | 17q11.1 | 25,283,930-25,462,376 | + |  |
|  |  | RP11-188G9 | 17q11.1 | 25,402,441-25,556,850 | + |  |
|  |  | **RP11-648N1** | 17q11.1 | 25,441,565-25,629,101 |  |  |
|  |  | **RP11-1057L7** | 17q11.1 | 25,527,383-25,734,185 |  |  |
|  |  | **RP11-1049N9** | 17q11.1-q11.2 | 25,719,008-25,912,180 |  |  |
|  |  | **RP11-803B22** | 17q11.2 | 25,887,028-26,087,281 |  |  |
|  |  | **RP11-909L10** | 17q11.2 | 26,018,836-26,201,098 |  |  |
|  |  | **RP11-59G20** | 17q11.2 | 26,152,884-26,322,729 |  |  |
|  |  | RP11-478B21 | 17q11.2 | 26,353,109-26,569,470 |  |  |
|  |  | RP11-398A1 | 17q11.2 | 29,853,490-30,046,575 |  |  |
|  |  | RP11-521P1 | 17q12 | 32,636,352-32,817,373 |  |  |
|  |  | RP11-294G4 | 17q12 | 33,327,055-33,497,797 |  |  |
|  | |  |  |  |  |  |
| **18p** | | RP11-16A21 | 18p11.21 | 12,440,267-12,620,284 |  |  |
|  |  | RP11-812N22 | 18p11.21 | 14,610,601-14,792,590 |  |  |
|  |  | **RP11-1025M21** | 18p11.21 | 14,884,393-15,063,720 | 2q21-q22, 21q11.1-q11.2 | 9p13-p12, 9q12-q13, 11q25, 21q21 |
|  |  | **RP11-749K13** | 18p11.21 | 15,104,134-15,308,545 | 2q21-q22, 7p12, 16p11.2, 21q11.1-q11.2 | 13q11-q12, 14q11.1-q11.2, 15q11.1-q11.2, 22q11.1-q11.2 |
|  |  | **RP11-1133K23** | 18p11.21-p11.1 | 15,272,044-15,409,381 | 2q21-q22, 9p12 | 13q11-q12, 14q11.1-q11.2, 15q11.1-q11.2, 21q11.1-q11.2, 22q11.1-q11.2 |
|  | |  |  |  |  |  |
| **18q** | | **RP11-1035O2** | 18q11.1 | 18,643,418-18,837,921 |  |  |
|  |  | **RP11-746M23** | 18q11.1-q11.2 | 18,839,613-19,023,838 |  |  |
|  |  | **RP11-10G8** | 18q11.2 | 19,020,441-19,177,003 |  |  |
|  |  | **CTD-3181O12** | 18q11.2 | 19,153,015-19,354,649 |  |  |
|  |  | **RP11-60G3** | 18q11.2 | 19,491,724-19,656,765 |  |  |
|  |  | RP11-90L7 | 18q11.2 | 23,355,089-23,520,954 |  |  |
|  |  |  |  |  |  |  |
| **19p** | | RP11-91L5 | 19p12 | 20,016,151-20,016,489 |  |  |
|  |  | RP11-22G10 | 19p12 | 23,179,046-23,328,665 |  |  |
|  |  | RP11-10G16 | 19p12 | 23,405,973-23,557,520 |  |  |
|  |  | **RP11-642I13** | 19p12 | 23,543,833-23,713,821 |  |  |
|  |  | **RP11-1151P6** | 19p12 | 23,783,598-23,940,464 |  |  |
|  |  | **RP11-698O1** | 19p12 | 23,896,161-24,051,285 |  |  |
|  |  | **RP11-460G17** | 19p12 | 24,054,312-24,211,459 |  |  |
|  |  | **CTD-3173A10** | 19p12 | 24,150,556-24,361,531 |  |  |
|  |  | **RP11-350E11** | 19p12-p11 | 24,350,566-24,557,363 |  |  |
|  | |  |  |  |  |  |
| **19q** | | RP11-775H18 | 19q11 | 27,732,071-27,934,662 |  |  |
|  |  | RP11-768C2 | 19q11 | 28,088,480-28,255,763 | + |  |
|  |  | **RP11-1058F6** | 19q11 | 28,208,919-28,403,934 |  |  |
|  |  | **CTD-2045N7** | 19q11q12 | 28,530,753-28,663,795 |  |  |
|  |  | **RP11-722H20** | 19q12 | 28,616,515-28,797,601 |  |  |
|  |  | **RP11-140G12** | 19q12 | 28,845,613-29,024,030 |  |  |
| **Tab. S1 Continued** | | | | | | |
| **19q** | | **RP11-983M7** | 19q12 | 29,028,596-29,220,852 |  |  |
|  |  | **RP11-347I6** | 19q12 | 29,346,657-29,551,645 |  |  |
|  |  | RP11-46I12 | 19q12 | 29,610,085-29,792,642 | + |  |
|  |  | RP11-79M11 | 19q12-q13.11 | 32,267,012-32,428,234 | + |  |
|  | |  |  |  |  |  |
| **20p** | | RP11-58B10 | 20p11.21 | 24,417,446-24,590,516 |  |  |
|  |  | RP11-79K14 | 20p11.21 | 24,868,862-25,026,986 |  |  |
|  |  | **RP11-156D15** | 20p11.21 | 25,105,245-25,287,374 |  |  |
|  |  | **RP11-922M14** | 20p11.21 | 25,287,371-25,453,834 |  |  |
|  |  | **RP11-147C9** | 20p11.21 | 25,413,329-25,588,554 |  |  |
|  |  | **RP11-161K13** | 20p11.21-p11.1 | 25,550,848-25,708,608 |  |  |
|  |  | **CTD-2011E20** | 20p11.1 | 25,691,534-25,845,105 | 7p12-p11, 9p12-p11, 17p11.2-p11.1 | 19q13.1, 21p11.2-p11.1 |
|  |  | **CTD-2514C3** | 20p11.1 | 25,946,248-26,067,566 | 22q11.2-q12 | 7p12-p11, 9p13-p12, 17p11.2-p11.1 |
|  |  | **CTD-2187D12** | 20p11.1 | 26,148,113-26,274,321 |  |  |
|  | |  |  |  |  |  |
| **20q*^c^*** | | **CTD-2311M18** | 20q11.21 | 29,420,721-29,548,171 | 9p12-p11, 13p11.2, 14p11.2, 15p11.2 | 7p12-p11, 9q12-q13, 12q12-q13 |
|  |  | **RP11-659L8** | 20q11.21 | 29,831,888-30,017,666 |  |  |
|  |  | **RP11-1070G8** | 20q11.21 | 30,076,682-30,266,629 |  |  |
|  |  | **RP11-1147I19** | 20q11.21 | 30,417,545-30,593,555 |  |  |
|  |  | RP11-483M19 | 20q11.21 | 30,610,396-30,792,581 |  |  |
|  |  | RP11-94H15 | 20q11.21-q11.22 | 31,921,665-32,106,833 |  |  |
|  |  | RP11-308J23 | 20q11.22 | 32,216,912-32,396,349 |  |  |
|  | |  |  |  |  |  |
| **21q*^c^*** | | **RP11-203F20** | 21q11.2 | 14,433,892-14,601,409 | 13q11-q12 |  |
|  |  | **RP11-79H3** | 21q11.2 | 14,535,068-14,535,581 | 13q11-q12 |  |
|  |  | **RP11-818G3** | 21q11.2 | 14,739,315-14,946,629 | 14q11.1-q11.2, 18p11.2-p11.1 |  |
|  |  | **RP11-72P4** | 21q11.2 | 14,935,928-15,120,351 | 2q21, 14q11.1-q11.2, 15q11.1-q11.2, 18p11.2-p11.1 | 13q11-q12, 22q11.1-q11.2 |
|  |  | **RP11-1025M7** | 21q11.2 | 15,154,895-15,367,989 | 2q11.1-q11.2, 4p12-p11, 9p12-p11 (ds), 9q13 (ds), 13p11.2-p11.1, 14p11.2-p11.1, 15p11.2-p11.1, 15q11.1-q11.2, 21p11.2-p11.1, 22p11.2-p11.1 |  |
|  |  | CTD-2379D11 | 21q11.2 | 15,330,140-15,458,985 | + |  |
|  |  | RP11-92E10 | 21q11.2 | 15,461,765-15,640,213 |  |  |
|  |  | RP11-31B6 | 21q11.2 | 16,076,691-16,249,383 |  |  |
|  |  | RP11-61A21 | 21q21.1 | 16,378,209-16,531,843 |  |  |
|  |  | RP11-141K11 | 21q21.1 | 16,669,300-16,845,906 |  |  |
|  | |  |  |  |  |  |
| **22q** | | RP11-112J20 | 22q11.1 | 16,132,286-16,319,155 | + |  |
|  |  | RP11-354F21 | 22q11.1 | 16,267,729-16,457,544 | + |  |
|  |  | RP11-134C5 | 22q11.1 | 16,379,229-16,519,909 | + |  |
|  |  | RP11-909J8 | 22q11.1 | 16,933,075-17,122,380 | + |  |
|  |  | RP11-164D11 | 22q11.1 | 16,969,076-17,154,240 | + |  |
|  |  | RP11-1057L20 | 22q11.1 | 17,154,241-17,336,721 |  |  |
|  |  | **RP11-66F9** | 22q11.1 | 17,381,569-17,556,908 |  |  |
|  |  | **RP11-155N18** | 22q11.1 | 17,567,626-17,725,595 |  |  |
|  |  | **RP11-958H20** | 22q11.1-11.21 | 17,853,653-18,040,246 |  |  |
|  |  | **RP11-143D17** | 22q11.21 | 17,971,412-18,136,104 |  |  |
|  |  | **RP11-91O6** | 22q11.21 | 18,146,104-18,330,474 |  |  |
|  |  | **RP11-18I11** | 22q11.21 | 18,266,921-18,419,003 |  |  |
|  | |  |  |  |  |  |
|  | |  |  |  |  |  |
| **Tab. S1 Continued** | |  |  |  |  |  |
| **22q** | | **RP11-81B3** | 22q11.21 | 18,417,690-18,609,914 |  |  |
|  |  | RP11-1053O2 | 22q11.21 | 18,679,974-18,895,750 | + |  |
|  |  | RP11-690P21 | 22q11.21 | 18,815,001-19,012,290 |  |  |
|  |  | RP11-71J20 | 22q11.21 | 19,068,436-19,259,741 |  |  |
|  |  | RP11-1057H19 | 22q11.21 | 19,310,701-19,484,643 |  |  |
|  |  | RP11-22M5 | 22q11.21 | 22,239,761-22,394,994 |  |  |
|  | |  |  |  |  |  |
| **Xp** | | RP11-145A8 | Xp11.21 | 54,994,332-55,156,028 |  |  |
|  |  | RP11-167P23 | Xp11.21 | 55,643,965-55,792,154 |  |  |
|  |  | CTD-3197N15 | Xp11.21 | 56,215,211-56,369,959 |  |  |
|  |  | RP11-465B24 | Xp11.21 | 56,448,804-56,615,904 |  |  |
|  |  | RP11-431N15 | Xp11.21 | 56,554,437-56,734,940 |  |  |
|  |  | RP11-1149D5 | Xp11.21 | 56,711,837-56,886,269 |  |  |
|  |  | CTD-3153O3 | Xp11.21 | 56,856,411-57,028,742 |  |  |
|  |  | RP11-825G4 | Xp11.21 | 56,955,154-57,135,177 |  |  |
|  |  | **RP11-936C8** | Xp11.21 | 57,145,040-57,321,580 |  |  |
|  |  | **RP11-1061F23** | Xp11.21 | 57,288,051-57,451,067 |  |  |
|  |  | **RP11-433F14** | Xp11.21 | 57,454,580-57,675,582 | 8q11.1-q11.2, 17p11.1-q11.2 |  |
|  |  | **CTD-2288B3** | Xp11.21 | 57,738,716-57,841,647 | 1p36.1-p35 |  |
|  |  | **CTD-2225J11** | Xp11.21-p11.1 | 57,986,852-58,100,452 |  |  |
|  | |  |  |  |  |  |
| **Xq** | | RP11-78G20 | Xq11.1 | 62,190,720-62,350,089 |  |  |
|  |  | RP11-841J22 | Xq11.1 | 62,504,474-62,674,269 |  |  |
|  |  | **RP11-90N17** | Xq11.1 | 62,593,405-62,753,538 |  |  |
|  |  | **RP11-977E19** | Xq11.1 | 62,712,198-62,889,534 |  |  |
|  |  | **RP11-943J20** | Xq11.1q11.2 | 62,833,058-63,012,869 |  |  |
|  |  | **RP11-151A2** | Xq11.1q11.2 | 62,959,394-63,124,490 | 19q12 |  |
|  |  | **RP11-594N5** | Xq11.2 | 63,291,045-63,462,303 |  |  |
|  |  | **RP11-284B18** | Xq11.2 | 63,434,365-63,538,898 |  |  |
|  |  | RP11-615J8 | Xq11.2 | 63,484,395-63,663,356 |  |  |
|  |  | RP11-185I15 | Xq11.2 | 64,082,296-64,259,949 |  |  |
|  |  | RP11-151G12 | Xq11.2-q12 | 64,504,716-64,671,259 | + |  |
|  |  | RP11-147G1 | Xq11.2 | 64,588,183-64,588,717 |  |  |
|  |  | RP11-368D24 | Xq12 | 64,788,640-64,988,641 |  |  |
|  |  | RP11-434B14 | Xq12 | 64,894,995-65,073,103 | + |  |
|  |  | CTD-2230P10 | Xq12 | 65,020,115-65,140,293 |  |  |
|  |  | CTD-3172H4 | Xq12 | 65,098,999-65,310,021 |  |  |
|  |  | RP11-607B14 | Xq12 | 65,418,956-65,564,261 |  |  |
|  |  | RP11-579G12 | Xq12 | 67,147,112-67,306,431 |  |  |
|  |  | RP11-76N1 | Xq12 | 67,265,258-67,410,694 |  |  |
|  | |  |  |  |  |  |
| **Yp*^e^*** | | RP11-145J12 | Yp11.2 | 3,955,463-4,104,601 |  |  |
|  |  | RP11-115H13 | Yp11.2 | 6,692,454-6,859,727 |  |  |
|  |  | RP11-45P11 | Yp11.2 | 8,733,894-8,895,637 |  |  |
|  |  | RP11-690A20 | Yp11.2 | 8,985,907-9,155,149 | + |  |
|  |  | RP11-693P14 | Yp11.2 | 9,461,460-9,634,529 | + |  |
|  |  | RP11-108I14 | Yp11.2 | 9,943,938-10,104,553 | +, most proximal available probe |  |
|  | |  |  |  |  |  |
|  | |  |  |  |  |  |
| **Tab. S1 Continued** | |  |  |  |  |  |
| **Yq** | | **RP11-1100G7** | Yq11.1-q11.21 | 13,278,562-13,454,479 | 1q11-q12, 13p13, 13p11.2, 14p13, 14p11.2, 15p13, 15p11.2, 16q12, 21p13, 21p11.2, 22p13, 22p11.2 | 3p13, 3q11.2-q12, 4p12-p11, 9q12-q13, 9q34 |
|  |  | **RP11-910C6** | Yq11.21 | 13,445,767-13,619,346 | 1q12-q21, 13p13, 13p11.2, 13q11q12, 16p11.2, 22p13, 22p11.2, 22q11.1-q11.2 | 14p13, 14p11.2, 14q11.1-q11.2, 15p13, 15p11.2, 15q11.1-q11.2, 21p13, 21p11.2, 21q11.1-q11.2 |
|  |  | **CTD-3080L23** | Yq11.21 | 13,824,298-13,968,689 | 1q43-q44 |  |
|  |  | **RP11-347B11** | Yq11.21 | 13,953,869-14,126,432 |  |  |
|  |  | **CTD-2657K20** | Yq11.21 | 14,138,449-14,344,226 |  |  |
|  |  | **RP11-91N9** | Yq11.21 | 14,342,539-14,503,826 |  |  |
|  |  | RP11-125B2 | Yq11.21 | 14,758,877-14,921,292 |  |  |
|  |  | CTD-2291B15 | Yq11.21 | 14,911,245-15,016,308 |  |  |
|  |  | RP11-113K10 | Yq11.21-q11.221 | 15,037,328-15,227,068 |  |  |
|  |  | RP11-71M14 | Yq11.221 | 16,664,046-16,664,205 |  |  |

*^a^*Probe physical localization is based on the hg19 human genome assembly.

*^b^*Cross-hybridizations are indicated in detail only for the probes belonging to the core panels (in bold).

*^c^*Core panel is incomplete and does not cover the heterochromatin/euchromatin bridge, because of the lack of a complete physical map in the reference sequence (chromosomes 1p, 10q, 11p, 20q, 21q) or the presence of paralogous segmental duplications, in which case the corresponding core panel probes were excluded from the clone set because their hybridization signals do not give unique mapping information (chromosomes 1q and 9p).

*^d^*Physical position of the clones mapped by FISH was considered, according to the UCSC Genome Browser Database.

***^e^***Physical map of the core panel does not exist in the reference sequence.

ds, double signal, +, present.
