# Supplementary material for: Design and validation of a pericentromeric BAC clone set aimed at improving diagnosis and phenotype prediction of supernumerary marker chromosomes
Source: Mol Cytogenet. 2013 Oct 30;6:45. doi: 10.1186/1755-8166-6-45 (PMC4176193; doi:10.1186/1755-8166-6-45)
Supplement: Additional file 2: Table S2 — Summary of 19 sSMCs characterized using the pericentromeric BAC clone set, including clinical data of the carrying patients. [file 1755-8166-6-45-S2.doc]

**Table S2** Summary of 19 sSMCs characterized using the pericentromeric BAC clone set.

| **Patient** | **Gender** | | **Sample** | **Origin** | **Conventional karyotype** | **Molecular-cytogenetic characterization** | | | **Pericentromeric noncritical regions*^b^*** | **Clinical signs** |
| --- | --- | --- | --- | --- | --- | --- | --- | --- | --- | --- |
|  |  | |  |  |  | **FISH and array CGH analyses** | **Breakpoint position** | **Euchromatic content (Mb*)^a^*** |  |  |
| 1 | F | | PBL | *De novo* | 47,XX,+mar[?]/46,XX[?] | ish der(1)(:p12?🡪q10:)  (wcp1+,RP11-320L8+,RP11-671M21+,  D1Z7/D5Z2/D19Z3+,D1Z1-) | 1p12 region  distal probe on sSMC RP11-320L8  (chr1:120,356,302–120,564,207)  bkp at less than ~120.36 Mb from 1p tel | Euchromatin 1p:  at least 1.14 Mb  (chr1: 120,356,302–121,500,001) | 115.9–142.6 Mb  from 1p tel | NA |
|  |  | |  |  |  |  |  |  |  |  |
| 2*^c^* | F | | PBL | *De novo* | 46,XX[150]/47,XX,+mar[41] | ish r(1)(::p12🡪q21.1::)  (RP11-320L8-,RP11-125P23-,  CTD-3138A9-,RP11-671M21+,  D1Z7/D5Z2/D19Z3+,CTD-2326L14-) | 1p12 region  bkp between BAC probes CTD-3138A9 and RP11-671M21 (chr1:120,696,242–120,747,163)  ~120.7 Mb < bkp < ~120.75 Mb from 1p tel  1q21.1 region  distal probe absent on sSRC CTD-2326L14  (chr1:144,507,758–144,822,788)  bkp at less than ~144.51 Mb from 1p tel | Euchromatin 1p:  min ~753 kb  (chr1: 120,747,163–121,500,001)  max ~804 kb  (chr1: 120,696,242–121,500,001)  Euchromatin 1q:  max ~1.91 Mb  (chr1:142,600,000–144,507,758) | 115.9–142.6 Mb  from 1p tel | Facial dysmorphism,  drug-resistant focal  epilepsy, mental  retardation |
|  |  | |  |  |  |  |  |  |  |  |
| 3*^d^* | M | | PBL | *De novo* | 46,XY[27]/47,XY,+mar[23] | ish r(4)(::p12🡪q13.1::)  (RP11-89F4-,RP11-178N2+,  RP11-90F11+,RP11-809H21+,  RP11-665L9+,RP11-192F3+,D4Z1+,  RP11-98B6+,RP11-796H19+,  RP11-41N7+,RP11-89B16+,  RP11-345F18+,RP11-474J22+,  RP11-91C3+,RP11-63E13-)  arr 4p12q13.1(45,754,992-61,126,608)x3 | 4p12 region  bkp between oligo A_14_P127561 and BAC RP11-178N2 (chr4:45,518,972–45,736,237)  at ~45.6 Mb from 4p tel  4q13.1 region  bkp between oligonucleotide probes A_14_P137150 and A_14_P133582 (chr4:61,126,608–61,343,360).  at ~61.2 Mb from 4p tel | Euchromatin 4p:  min 2.5 Mb  (chr4:45,736,237–48,200,001)  max 2.7 Mb  (chr4:45,518,972–48,200,001)  Euchromatin 4q:  min 8.4 Mb  (chr4:52,700,000–61,126,608)  max ~8.6 Mb  (chr4:52,700,000–61,343,360) | 44.13–55.03(?) or 62.80 (?) Mb  from 4p tel | Asthenoteratozoospermia  ascertained during testing before *in vitro* fertilization |
|  |  | |  |  |  |  |  |  |  |  |
| 4*^c^* | F | | AF | *De novo* | 46,XX[7]/47,XX,+mar[3]  Postnatal cytogenetic analysis in PBL confirmed very low sSMC(10) mosaicism | ish der(10)(p11.23?🡪q11.21)  (RP11-39E10+,RP11-188L14+,  CTD-2530C6+,D10Z1+,RP11-178A10dim,  RP11-351D16-,RP11-42B19-,  RP11-71N21-) | 10p11.23 region  distal probe on sSMC RP11-39E10  (chr10:31,143,313–31,323,431)  bkp at less than ~31.14 Mb from 10p tel  10q11.21 region  RP11-178A10 interrupted  (chr10:42,877,687–43,000,769)  ~42.9 Mb < bkp > 43 Mb from 10p tel | Euchromatin 10p:  at least 6.86 Mb  (chr10:31,143,313–38,000,001)  Euchromatin 10q:  max ~0.7 Mb  (chr10:42,300,001–43,000,769) | 34.72–44.51 Mb  from 10p tel | Pre-term delivery,  failure to thrive,  neonatal hypotonia,  dysmorphism |
| **Tab. S2 Continued** | | | | | | | | | | |
| 5*^d^* | | M | PBL | *De novo* | 47,XY,+mar[86]/46,XY[14] | ish r(11)(::p11.12🡪q13.1::)  (RP11-1062A8-,RP11-746P9+,  RP11-806K4+,RP11-318O24+,  RP11-685O18+,RP11-100E23+,  D11Z1+,RP11-236L12+,  RP11-352C11+,RP11-872D17+)  arr 11p11.12q13.1(50,378,743-63,743,029)x3 | 11p11.12 region  bkp between oligo A_14_P126799 and BAC  RP11-746P9 (chr11:49,919,441–50,111,641)  at ~50 Mb from 11p tel  11q13.1 region  bkp between oligo A_14_P138783 and A_14_P129860  (chr11:63,743,029–63,756,165)  at ~63.7 Mb from 11p tel | Euchromatin 11p:  min 1.5 Mb  (chr11:50,111,641–51,600,001)  max 1.7 Mb  (chr11:49,919,441–51,600,001)  Euchromatin 11q:  min ~10.04 Mb  (chr11:53,700,001–63,743,029)  max ~10.06 Mb  (chr11:53,700,001–63,756,165) | 50.95-60.43Mb  from 11p tel | Born at term from a twin pregnancy  by caesarean section.  The twin sister was healthy.  At birth: weigh 2,360 g (3^rd^ percentile), length (3^rd^) and OFC (3^rd^). Apgar scores 10/10, neonatal hypocalcemia. At 15 months normal growth parameters, psychomotor retardation (no walking and speech) and dysmorphic feature: dysmorphic ears, short palpebral fissures, long philtrum, tapering fingers. |
|  | |  |  |  |  |  |  |  |  |  |
| 6*^e^* | | F | PBL | Maternal | 47,XX,+mar | ish der(13)(pter🡪q11::q11🡪pter)  (D13Z1/D21Z1+,RP11-79H3-,  CTD-3188C8-,RP11-294G16-,RP11-301J16-)  or der(21)  (pter🡪q11.1~q11.2::q11.1~q11.2🡪pter)  (D13Z1/D21Z1+,RP11-203F20-,  RP11-79H3-) | bkps at chr 13 centromere  or  21q11.1~q11.2 region  first distal probe absent on sSMC  RP11-203F20  (chr21:14,433,892–14,601,409)  bkps at ~14.3-14.43 Mb from 21p tel | Chr 13q heterochromatin  or  Euchromatin 21q:  if present max ~134 kb  (chr21:14,300,000–14,433,892) | Up to 17.95 Mb  from 21p tel | No clinical signs |
|  | |  |  |  |  |  |  |  |  |  |
| 7*^c^* | | F | PBL | *De novo* | 47,XX,+mar | ish der(14)  (pter🡪 q11.1~q11.2::q11.1~q11.2🡪pter)  (D14Z1/D22Z1+,RP11-134C5-,  RP11-112J20-)  or der(22)(pter🡪q11.1::q11.1🡪pter)  (D14Z1/D22Z1+,RP11-112J20-,  RP11-134C5-,RP11-1057L20-,RP11-91O6-) | 14q11.1~q11.2 region  first distal probe absent on sSMC  RP11-134C5  (chr14:19,307,335–19,454,103)  bkps at ~19.1-19.3 Mb from 14p tel  or  bkps at chr 22 centromere | Euchromatin 14q:  if present max ~207 kb  (chr14:19,100,000–19,307,335)  or  Chr 22q heterochromatin | Up to 20.24 Mb  from 14p tel | Growth delay |
|  | |  |  |  |  |  |  |  |  |  |
|  | | | | | | | | | | |
|  | | | | | | | | | | |
|  | | | | | | | | | | |
|  | | | | | | | | | | |
|  | | | | | | | | | | |
|  | | | | | | | | | | |
| **Tab. S2 Continued** | | | | | | | | | | |
| 8*^c^* | | M | AF | Maternal | 47,XY,+mar | ish der(14)  (pter🡪 q11.1~q11.2::q11.1~q11.2🡪pter)  (D14Z1/D22Z1+,RP11-134C5-,  RP11-354F21-,RP11-831B15-)  or der(22)(pter🡪q11.1::q11.1🡪pter)  (D14Z1/D22Z1+,RP11-354F21-,  RP11-134C5-,RP11-958H20-) | 14q11.1~q11.2 region  first distal probe absent on sSMC  RP11-134C5  (chr14:19,307,335–19,454,103)  bkps at ~19.1–19.3 Mb from 14p tel  or  bkps at chr 22 centromere | Euchromatin 14q:  if present max ~207 kb  (chr14:19,100,000–19,307,335)  or  Chr 22q heterochromatin | Up to 20.24 Mb  from 14p tel | No clinical signs |
|  | |  |  |  |  |  |  |  |  |  |
| 9*^c^* | | F | AF | *De novo* | 47,XX,+mar | ish der(14)  (pter🡪 q11.1~q11.2::q11.1~q11.2🡪pter)  (D14Z1/D22Z1+,RP11-134C5-,  RP11-354F21-,RP11-831B15-)  or der(22)(pter🡪q11.1::q11.1🡪pter)  (D14Z1/D22Z1+,RP11-354F21-,  RP11-134C5-,RP11-958H20-) | 14q11.1~q11.2 region  first distal probe absent on sSMC  RP11-134C5  (chr14:19,307,335–19,454,103)  bkps at ~19.1–19.3 Mb from 14p tel  or  bkps at chr 22 centromere | Euchromatin 14q:  if present max ~207 kb  (chr14:19,100,000–19,307,335)  or  Chr 22q heterochromatin | Up to 20.24 Mb  from 14p tel | No clinical signs |
|  | |  |  |  |  |  |  |  |  |  |
| 10*^c^* | | F | PBL | Maternal balanced  t(6;14)(p25;q11.1) | 47,XX,+mar | ish der(14)t(6;14)(p25;q11.1~q11.2)  (D14Z1/D22Z1+,RP11-134C5-,  RP11-112J20-,RP11-354F21-,RP11-14J7-,6pter+) | 14q11.1~q11.2 region  first distal probe absent on sSMC  RP11-134C5  (chr14:19,307,335–19,454,103)  bkps at ~19.1–19.3 Mb from 14p tel | Euchromatin 6p25🡪pter  Euchromatin 14q:  if present max ~207 kb  (chr14:19,100,000–19,307,335) | Up to 20.24 Mb  from 14p tel | Uneventful  pregnancy; dysmorphic  features at birth |
|  | |  |  |  |  |  |  |  |  |  |
| 11*^c^* | | F | PBL | Maternal balanced  t(6;14)(p25;q11.1) | 47,XX,+mar | ish der(14)t(6;14)(p25;q11.1~q11.2)  (D14Z1/D22Z1+,RP11-134C5-,  RP11-112J20-,RP11-354F21-,RP11-14J7-,6pter+) | 14q11.1~q11.2 region  first distal probe absent on sSMC  RP11-134C5  (chr14:19,307,335–19,454,103)  bkps at ~19.1–19.3 Mb from 14p tel | Euchromatin 6p25🡪pter  Euchromatin 14q:  if present max ~207 kb  (chr14:19,100,000–19,307,335) | Up to 20.24 Mb  from 14p tel | Uneventful  pregnancy;  dysmorphic features  and skeletal  malformations at birth |
|  | |  |  |  |  |  |  |  |  |  |
| 12 | | M | AF | Maternal | 47,XY,+mar | ish idic(15;15)(pter🡪q11.2::q11.2🡪pter)  (wcp15-,D15Z4++,RP11-357P4++,  RP11-11H9++,RP11-415B7++,  RP11-467N20dim,SNRPN-,D15S10-) | 15q11.2 region  RP11-467N20 interrupted  (chr15:22,646,319–22,830,873)  bkps at ~22.7 Mb from 15p tel | Euchromatin 15q:  min ~1.9 Mb  (chr15:20,700,000–22,646,319)  max ~2.1 Mb  (chr15:20,700,000–22,830,873) | Up to 23.70 Mb  from 15p tel | Normal fetal growth  parameters |
|  | |  |  |  |  |  |  |  |  |  |
|  | | | | | | | | | | |
|  | | | | | | | | | | |
|  | | | | | | | | | | |
| **Tab. S2 Continued** | | | | | | | | | | |
| 13 | | F | PBL | Familial | 47,XX,+mar | ish idic(15;15)(pter🡪q11.2::q11.2🡪pter) (D15Z4++,RP11-357P4++,RP11-11H9++,  RP11-415B7++,RP11-467N20dim,  SNRPN-) | 15q11.2 region  RP11-467N20 interrupted  (chr15:22,646,319–22,830,873)  bkps at ~22.7 Mb from 15p tel | Euchromatin 15q:  min ~1.9 Mb  (chr15:20,700,000–22,646,319)  max ~2.1 Mb  (chr15:20,700,000–22,830,873) | Up to 23.70 Mb  from 15p tel | No clinical signs |
|  | |  |  |  |  |  |  |  |  |  |
| 14 | | F | AF | *De novo* | 47,,XX,+mar | ish r(16)(::p11.1🡪q11::)(RP11-1088B6-,  RP11-488I20-,D16Z1+,CTD-2382P11-,  RP11-627O2-,RP11-671L23-) | bkp1 at chromosome 16p11.1  bkp2 at 16q11.1–q11.2 region  at less than 46.64 Mb from 16p tel | Exclusively heterochromatin | 28.96–47.50 Mb  from 16p tel | Phenotypically  normal child  at birth; at subsequent follow-up normal psychomotor development |
|  | |  |  |  |  |  |  |  |  |  |
| 15 | | F | PBL | *De novo* | 47,XX,+mar1/  48,XX,+mar1,+mar2[?]/  49,XX,+mar1,+mar2,+mar3[?] | mar1:  idic(18;18)(p11.21🡪q10::q10🡪p11.21)  (RP11-1025M21dim,RP11-749K13++,  RP11-1133K23++,D18Z1++,RP11-1035O2-)  mar2:  ish der(2)(:p11.1?🡪q11.1:)  (RP11-165D20+,RP11-1069D4+,  CTD-2182L24+,D2Z1+,RP11-134N21-,  RP11-71B7+,RP11-708D7-)  mar3? | mar1  18p11.21 region  RP11-1025M21 interrupted  (chr18:14,884,393–15,063,720)  bkps at ~15.0 Mb from 18p tel  mar2  bkp1at 2p not completely characterized  bkp2 at chromosome 2q11.1 | Euchromatin 18p:  min ~516 kb  (chr18:14,884,393–15,400,001)  max 336 kb  (chr18:15,063,720–15,400,001)  Euchromatin 2p? | 12.80–19.87 Mb  from 18p tel  85.22–102.22 Mb  from 2p tel | No clinical signs |
|  | |  |  |  |  |  |  |  |  |  |
| 16 | | F | AF | Unknown | 47,XX,+mar | ish idic(22;22)(pter🡪q11.21::q11.21🡪pter)  (D14Z1/D22Z1++,RP11-91O6++,  RP11-81B3++,RP11-1053O2dim,  RP11-1057H19-,D22S553-,D22S609-,  D22S942-) | 22q11.21 region  RP11-1053O2 interrupted  (chr22:18,679,974–18,895,750)  bkps at ~18.8 Mb from 22p tel | Euchromatin 22q:  min ~780 kb  (chr22:17,900,000-18,679,974)  max ~996 kb  (chr22:17,900,000–18,895,750) | Up to18.00 Mb  from 22p tel | NA |
|  | |  |  |  |  |  |  |  |  |  |
| 17 | | M | PBL | *De novo* | 47,XY,+mar | ish idic(22;22)(pter🡪q11.21::q11.21🡪pter)  (D14Z1/D22Z1++,RP11-91O6++,  RP11-81B3++,RP11-1053O2++,  RP11-690P21dim,RP11-1057H19-,  D22S553-,D22S609-,D22S942-) | 22q11.21 region  RP11-690P21 interrupted  (chr22:18,815,001–19,012,290)  bkps at ~18.9 Mb from 22p tel | Euchromatin 22q:  min ~915 kb  (chr22:17,900,000–18,815,001)  max ~1.1 Mb  (chr22:17,900,000–19,012,290) | Up to18.00 Mb  from 22p tel | Cat eye syndrome:  dysmorphic features,  coloboma, psychomotor development delay |
|  | |  |  |  |  |  |  |  |  |  |
|  | |  |  |  |  |  |  |  |  |  |
| **Tab. S2 Continued** | | | | | | | | | | |
| 18 | | M | PBL | *De novo* | 46,XY[37]/47,XY,+mar[13] | idic(22;22)(pter🡪q11.21::q11.21🡪pter)  (D14Z1/D22Z1++,RP11-1112K21++,  RP11-958H20++,RP11-81B3++,  RP11-1053O2+,RP11-690P21dim,  RP11-71J20-,RP11-22M5-,HIRA-) | 22q11.21 region  bkp1 between RP11-81B3 and RP11-1053O2  (chr22:18,609,914–18,679,974)  at ~18.65 Mb from 22p tel  RP11-690P21 interrupted  (chr22:18,815,001–19,012,290)  bkp2 at ~18.9 Mb from 22p tel | Euchromatin 22q:  tetrasomy of min ~710 kb  (chr22:17,900,000–18,609,914)  max ~780 kb  (chr22:17,900,000–18,679,974)trisomy of min ~135 kb  (chr22: 18,679,974–18,815,001)  max ~402 kb  (chr22: 18,609,914–19,012,290) | Up to18.00 Mb  from 22p tel | At 6 months normal psychomotor  development. No dysmorphic features except for face  asymmetry and reduced  right eyelid |
| *^a^*The amount of euchromatic DNA comprising each marker is based on the chromosomal position of the most distal pericentromeric BAC clone present on the sSMC and the start of the known euchromatic sequence, both from the human genome assembly hg19 of the UCSC Genome Browser Database, released February 2009.  *^b^*Chromosomal position of the possible pericentromeric uncritical regions based on human genome assembly hg19, available on the sSMC database at <http://www.fish.uniklinikum-jena.de/sSMC.html>.  *^c^*In patients 2, 4, and 7–11 the sSMC characterization was not completed because of sample insufficiency.  *^d^*In patients 3 and 5, the sSMC characterization was completed by array CGH analysis, and the amount of euchromatic content was estimated by considering the physical position of the most distal pericentromeric BAC clone present on the sSMC, as well as the most distal duplicated and non-duplicated oligonucleotides on the 4×44K Agilent array.  *^e^*In patient 6, the sSMC characterization was not completed because of the lack of a complete pericentromeric physical map of 21q in the reference sequence .  AF, amniotic fluid; bkp, breakpoint; F, female; M, male; NA, not available; PBL, peripheral blood lymphocytes; tel, telomere. | | | | | | | | | | |
